# RFX3 is essential for the generation of functional human pancreatic islets from stem cells

**DOI:** 10.1007/s00125-025-06424-4

**Published:** 2025-04-23

**Authors:** Bushra Memon, Noura Aldous, Ahmed K. Elsayed, Sadaf Ijaz, Sikander Hayat, Essam M. Abdelalim

**Affiliations:** 1 Diabetes Research Center, Qatar Biomedical Research Institute (QBRI), Hamad Bin Khalifa University (HBKU), Qatar Foundation (QF), Doha, Qatar; 2 Pluripotent Stem Cell Disease Modeling Lab, Translational Medicine Department, Research Branch, Sidra Medicine, Doha, Qatar; 3 College of Health and Life Sciences, Hamad Bin Khalifa University (HBKU), Qatar Foundation (QF), Doha, Qatar; 4 Department of Medicine 2 (Nephrology, Rheumatology, Clinical Immunology and Hypertension), Medical Faculty, RWTH Aachen University, Aachen, Germany

**Keywords:** Differentiation, Endocrine pancreas, Enterochromaffin cells, iPSC model, Transcription factor

## Abstract

**Aims/hypothesis:**

The role of regulatory factor X 3 (RFX3) in human pancreatic islet development has not been explored. This study aims to investigate the function of RFX3 in human pancreatic islet development using human islet organoids derived from induced pluripotent stem cells (iPSCs), hypothesising that RFX3 regulates human islet cell differentiation.

**Methods:**

We generated *RFX3* knockout (*RFX3* KO) iPSC lines using CRISPR/Cas9 and differentiated them into pancreatic islet organoids. Various techniques were employed to assess gene expression, cell markers, apoptosis, proliferation and glucose-stimulated insulin secretion. Single-cell RNA-seq datasets from human embryonic stem cell-derived pancreatic islet differentiation were re-analysed to investigate *RFX3* expression in specific cell populations at various developmental stages. Furthermore, bulk RNA-seq was conducted to further assess transcriptomic changes. RFX3 overexpression was implemented to reverse dysregulated gene expression.

**Results:**

RFX3 was found to be highly expressed in pancreatic endocrine cell populations within pancreatic progenitors (PPs), endocrine progenitors (EPs) and mature islet stages derived from iPSCs. Single-cell RNA-seq further confirmed RFX3 expression across different endocrine cell clusters during differentiation. The loss of *RFX3* disrupted pancreatic endocrine gene regulation, reduced the number of hormone-secreting islet cells and impaired beta cell function and insulin secretion. Despite a significant reduction in the expression levels of pancreatic islet hormones, the pan-endocrine marker chromogranin A remained unchanged at both EP and islet stages, likely due to an increase in the abundance of enterochromaffin cells (ECs). This was supported by our findings of high EC marker expression levels in *RFX3* KO EPs and islets. In addition, *RFX3* loss led to smaller islet organoids, elevated thioredoxin-interacting protein levels and increased apoptosis in EPs and islets. Furthermore, *RFX3* overexpression rescued the expression of dysregulated genes in *RFX3* KO at the PP and EP stages.

**Conclusions/interpretation:**

These findings underscore the crucial role of RFX3 in regulating human islet cell differentiation and its role in suppressing EC specification. These insights into RFX3 function have implications for understanding islet biology and potential diabetes susceptibility.

**Data availability:**

The RNA-seq datasets have been submitted to the Zenodo repository and can be accessed via the following links: DOI 10.5281/zenodo.13647651 (PPs); and DOI 10.5281/zenodo.13762055 (SC-islets).

**Graphical Abstract:**

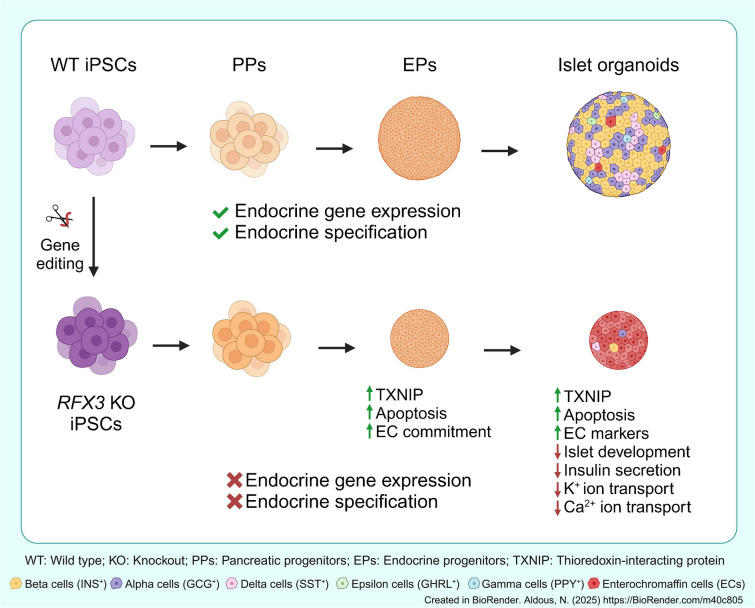

**Supplementary Information:**

The online version contains peer-reviewed but unedited supplementary material available at 10.1007/s00125-025-06424-4.


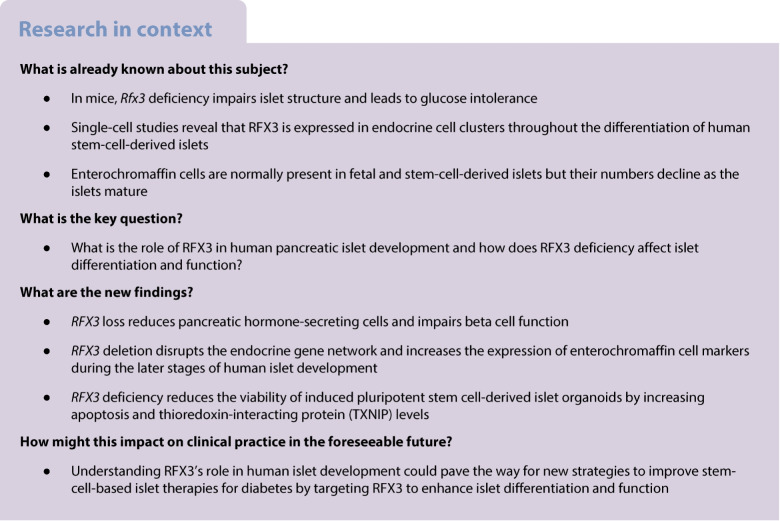


## Introduction

The pancreatic islets regulate blood glucose levels, and defects in their development or function are associated with diabetes. While animal studies provide insights into islet development and diabetes, significant differences in islet structure and endocrine cell physiology complicate modelling human diabetes [1]. Human pluripotent stem cells (hPSCs) provide a valuable tool for modelling diseases like diabetes [2, 3].

Regulatory factor X3 (RFX3) is implicated in pancreatic islet development in mice and is expressed in neurogenin 3 (NEUROG3)-positive endocrine progenitors (EPs) from embryonic day 13.5 and continuing in adult islets, along with insulin (INS), glucagon (GCG), somatostatin (SST) and pancreatic polypeptide Y (PPY) [4, 5]. In *Rfx3*^−/−^ mice, surviving embryos exhibit impaired islet cytoarchitecture with reduced numbers of INS^+^, GCG^+^ and SST^+^ cells, increased PPY^+^ cells and glucose intolerance. Despite these defects, *Rfx3* deficiency does not impact pancreatic developmental marker genes such as *Pdx1*, *Nkx6.1* (also known as *Nkx6-1*), *Neurod1* and *Mafa* [4, 5]. While another regulatory factor X family member, RFX6, is known to regulate human islet development [6–8] and mutations cause monogenic diabetes [9, 10], the role of RFX3 in human development remains unclear.

Given RFX3’s crucial role in rodent pancreatic islet cell development, investigating its function in humans is highly warranted. In this study, we generated an *RFX3* knockout (*RFX3* KO) induced pluripotent stem cell (iPSC) model to investigate the role of RFX3 in human pancreatic islet development.

## Methods

### hPSC maintenance and *RFX3* KO iPSC generation

iPSCs from a healthy individual were generated and fully characterised in our laboratory, as described in our previous report [11]. A CRISPR/Cas9 technique was used to generate *RFX3* KO iPSC lines from wild-type (WT) iPSCs. Two *RFX3* KO iPSC lines were used for all experiments (*RFX3* KO1 and *RFX3* KO2) (see electronic supplementary material [ESM] Methods for details).

### Differentiation of hPSCs into islet organoids

Differentiation began when hPSCs reached 70–80% confluency and continued until pancreatic progenitors (PPs) were formed, using our optimised protocol (ESM Fig. 1a) [12]. To drive beta cell differentiation, PPs were dissociated, re-aggregated in Aggrewell 400 24-well plates (STEMCELL Technologies, Vancouver, Canada) with PP media and cultured for 2 days [13]. Organoids were then transferred to ultra-low attachment plates in EP media on a shaker for maturation into islets (see ESM Methods, ESM Table 1 for details).

### Real-time quantitative PCR, western blotting, immunostaining and flow cytometry

Real-time quantitative PCR (RT-qPCR), western blotting, immunostaining and flow cytometry were performed as reported previously [8, 14] (see ESM Methods, ESM Tables 2, 3 for details).

### Apoptosis and proliferation assays

Apoptosis and proliferation assays were performed using Annexin V and BrdU incorporation assays, as previously described [8, 14] (see ESM Methods for details).

### Insulin secretion assays

Equal numbers of iPSC-derived islet organoids (SC-islets) were collected, washed with PBS and equilibrated in KRB with 2 mmol/l glucose for 2 h at 37°C, 5% CO_2_. They were then treated with low glucose (2 mmol/l), high glucose (20 mmol/l) or 30 mmol/l KCl in KRB for 30 min, and supernatant fractions collected. For mitochondrial substrate testing, after 2 h of preincubation in KRB with 2.8 mmol/l glucose, cells were treated with low glucose (2.8 mmol/l) with 10 mmol/l methyl pyruvate for 1 h at 37°C. Supernatant fractions were centrifuged, and the top 100 µl collected for insulin measurement using ELISA (RayBiotech, USA), normalised to total cell number.

For total insulin content, islet cells were washed with PBS, treated with 300 µl acid-ethanol solution (1.5% [vol./vol.] HCl in 70% [vol./vol.] ethanol), vortexed, and incubated for 48 h at −20°C, with vortexing after 24 h. After centrifugation at 2100 *g* for 15 min, the supernatant fraction was collected and analysed using an insulin ELISA assay (ALPCO, 80-INSHU-E01.1, USA). The pellet was dried at 80°C, resuspended in water and DNA was quantified. Insulin content was normalised to DNA concentration [15, 16].

### Single-cell RNA-seq and bulk RNA-seq analyses

The online published GSE202497 single-cell RNA-seq dataset for human embryonic stem cell (hESC) differentiation into pancreatic islets (https://www.ncbi.nlm.nih.gov/geo/query/acc.cgi?acc=GSE202497) was used [17] and re-analysed as previously described [8]. Furthermore, bulk RNA-seq results were analysed as previously described [8]. See ESM Methods for details.

### RFX3 overexpression

RFX3 overexpression was performed, as previously reported [14] (see ESM Methods for details).

### Statistical analysis

At least three biological replicates were analysed for each experiment, and *p* values were calculated using an ANOVA test on GraphPad Prism 8 software (GraphPad Software, Boston, MA, USA; www.graphpad.com). Data are represented as mean ± SD.

## Results

### RFX3 is highly expressed in stem-cell-derived pancreatic endocrine lineages

**Fig. 1:**
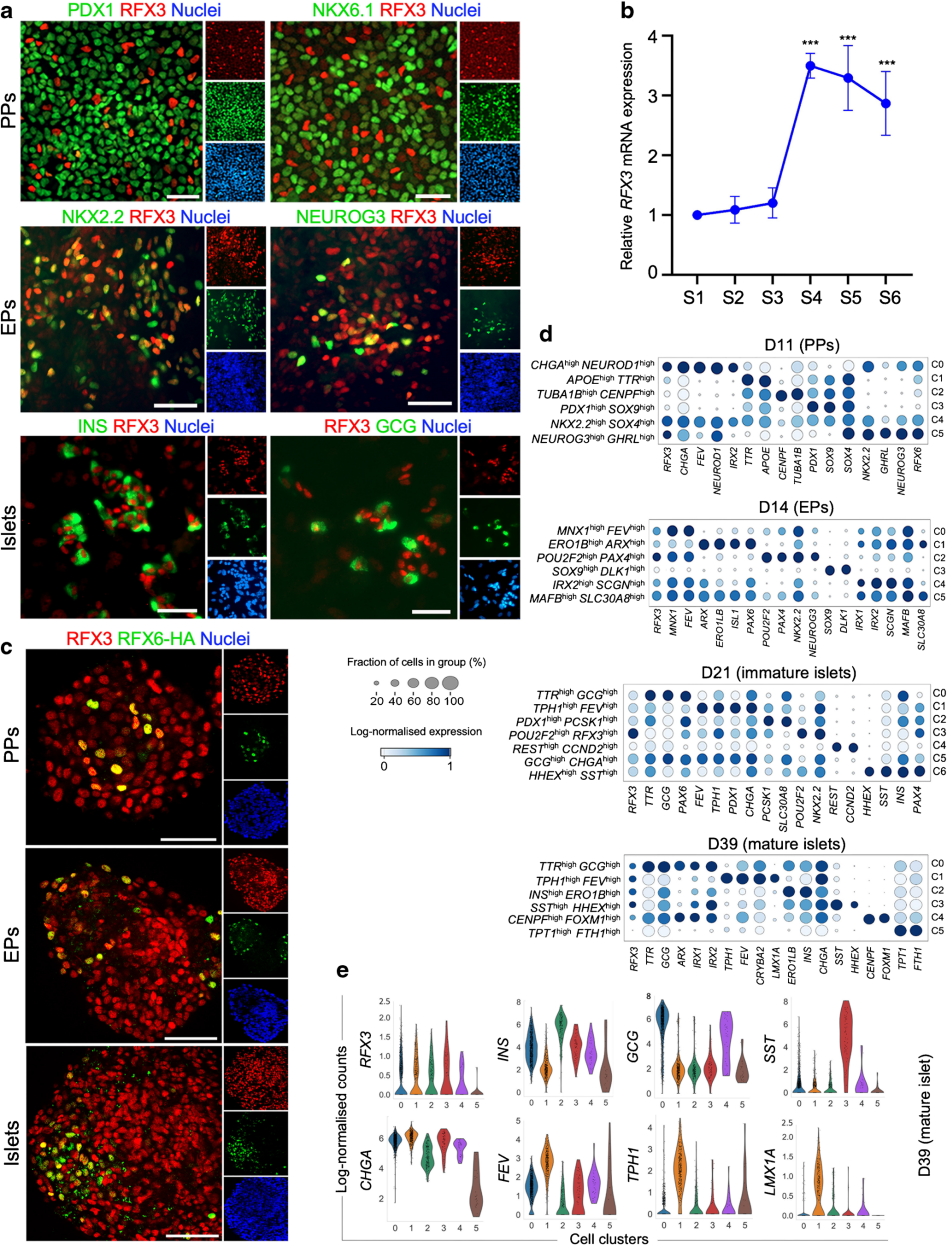
RFX3 is predominantly expressed in pancreatic endocrine lineages during differentiation of iPSCs into islets. (**a**) Immunofluorescence images showing stage-specific expression pattern of RFX3 in pancreatic cells (images representative of at least three biological experiments). Note the absence of RFX3 expression in PDX1^+^ and NKX6.1^+^ cells at the PP stage. RFX3 was co-expressed with NKX2.2 and NEUROG3 in iPSC-derived EPs and with INS and GCG in iPSC-derived islets. (**b**) Timeline RT-qPCR analysis for *RFX3* mRNA expression during pancreatic islet cell differentiation (*n*=4), from definitive endoderm (S1) to primitive gut tube (S2), posterior foregut (S3), PP (S4) EP (S5) and islet stages (S6). (**c**) Immunofluorescence images showing the co-expression of RFX3 and RFX6 in PPs, EPs and islets derived from hESC-H9 RFX6 HA-tagged cell line. (**d**) Dot plots demonstrating gene expression in various cell clusters determined by single-cell RNA-seq. The expression level in each cluster (C0–C5) is scaled based on the percentages of cells expressing *RFX3* (dot size) and mean expression (colour intensity) of the gene. Dot plots are presented for day 11 (D11), day 14 (D14), day 21 (D21) and day 39 (D39) of hESC differentiation. (**e**) Violin plots depicting expression pattern of key islet and EC markers across various cell clusters (as shown in **d**) at day 39 (mature islets). Data are presented as means±SD. ***p<0.001. Scale bar, 50 µm

To investigate the stage-specific role of RFX3 in human pancreatic islet development, we differentiated WT iPSCs into SC-islets, and RFX3 expression was evaluated at various stages of this differentiation (Fig. 1a, b and ESM Fig. 1). The expression of RFX3 was not detected during the definitive endoderm, primitive gut tube or posterior foregut stages of differentiation (ESM Fig. 1b). However, immunofluorescence analysis showed a high expression of RFX3 in PPs. Interestingly, RFX3 expression was notably high in PPs not expressing pancreatic and duodenal homeobox 1 (PDX1) or NK6 homeobox 1 (NKX6.1) (Fig. 1a). In iPSC-derived EPs, we observed co-localisation of RFX3 with the endocrine markers NK2 homeobox 2 (NKX2.2) and NEUROG3, and upon differentiation to islets all INS- and GCG-expressing cells showed nuclear expression of RFX3 (Fig. 1a). Furthermore, RT-qPCR analysis demonstrated that *RFX3* mRNA expression increased during endocrine differentiation, with the highest level being in PP, EP and islet stages (Fig. 1b). Co-expression analysis of RFX3 and RFX6 during pancreatic differentiation showed that all RFX6^+^ cells at the PP, EP and islet stages co-expressed RFX3, although RFX3 was also present in many RFX6^−^ cells (Fig. 1c).

To decipher the cell-type-specific role of RFX3 in pancreatic development, we re-analysed a single-cell RNA-seq dataset of hESC-derived islets, focused on time points for PPs, EPs, immature islets and mature islets (day 11, 14, 21 and 39, respectively) [17]. Cell clusters from unsupervised clustering were visualised using uniform manifold approximation and projection (UMAP) and assigned based on key mRNA expression signatures for each stage (Fig. 1d, e). At D11 (PPs), *RFX3* was strongly expressed in endocrine cell populations represented by a high expression of *CHGA*, *FEV*, *NEUROD1*, *NKX2.2* (also known as *NKX2-2*), *NEUROG3*, *RFX6*, *GHRL*, *IRX2* and *SOX4* within different clusters whereas low expression of *RFX3* was observed in multipotent progenitor cell populations represented by high expression of *PDX1* and *SOX9* (Fig. 1d and ESM Fig. 2). The lack of *RFX3* expression in the *PDX1*^high^ *SOX9* ^high^ cluster aligns with our immunostaining results showing low expression levels of RFX3 in PDX1^+^ and NKX6.1^+^ PPs (Fig. 1a). *RFX3* expression was also low in the *APOE*^high^ *TTR*^high^ and proliferative *TUBA1B*^high^ *CENPF*^high^ clusters (Fig. 1d). On day 14 (EPs), *RFX3* expression was observed in most clusters representing EP subpopulations, with the highest levels in the *POU2F2*^high^ *PAX4*^high^ cluster, which also showed high levels of *NEUROG3* and *NKX2.2* expression, and moderate levels of *MNX1* and *FEV* expression (Fig. 1d). Other endocrine markers that were highly expressed in clusters showing moderate *RFX3* levels were *ARX*, *ISL1*, *ERO1B, PAX6*, *SCGN*, *IRX1*, *IRX2*, *MAFB* and *SLC30A8*, while the lowest *RFX3* expression was in the *SOX9*^high^ *DLK1*^high^cluster (Fig. 1d). On day 21 (immature islets), the highest *RFX3* expression was observed in an endocrine cluster (*POU2F2*^high^ *RFX3*^high^), with moderate *RFX3* expression in other endocrine clusters (Fig. 1d). On day 39 (mature islets), *RFX3* was prominently expressed in distinct endocrine cell populations: *INS*^high^ *ERO1LB*^high^ (beta cells), *TTR*^high^ *GCG*^high^ (alpha cells), *SST*^high^ *HHEX*^high^ (delta cells) and *TPH1*^high^ *FEV*^high^ (enterochromaffin-like cells) (Fig. 1d, e). *RFX3* expression was low in the *CENPF*^high^ *FOXM1*^high^ (proliferation) cluster and negligible in the *TPT1*^high^ *FTH1*^high^ (ribosome biogenesis) cluster (Fig. 1d). Interestingly, we found that all *RFX6*-expressing clusters also expressed *RFX3*, with some variation in *RFX3* levels across endocrine clusters at different stages (ESM Fig. 2a, b), consistent with the immunostaining results. In contrast, clusters expressing known exocrine markers (*PTF1A*, *CPA1* and *CPA2*) did not show *RFX3* expression (ESM Fig. 2c), as confirmed by the lack of co-localisation with the exocrine marker chymotrypsin (ESM Fig. 2d). These results suggest that *RFX3* is expressed during progenitor differentiation and endocrine specification, and in hormone-expressing mature islets.

### *RFX3* deletion specifically impairs the endocrine gene regulatory network in iPSC-derived PPs

**Fig. 2:**
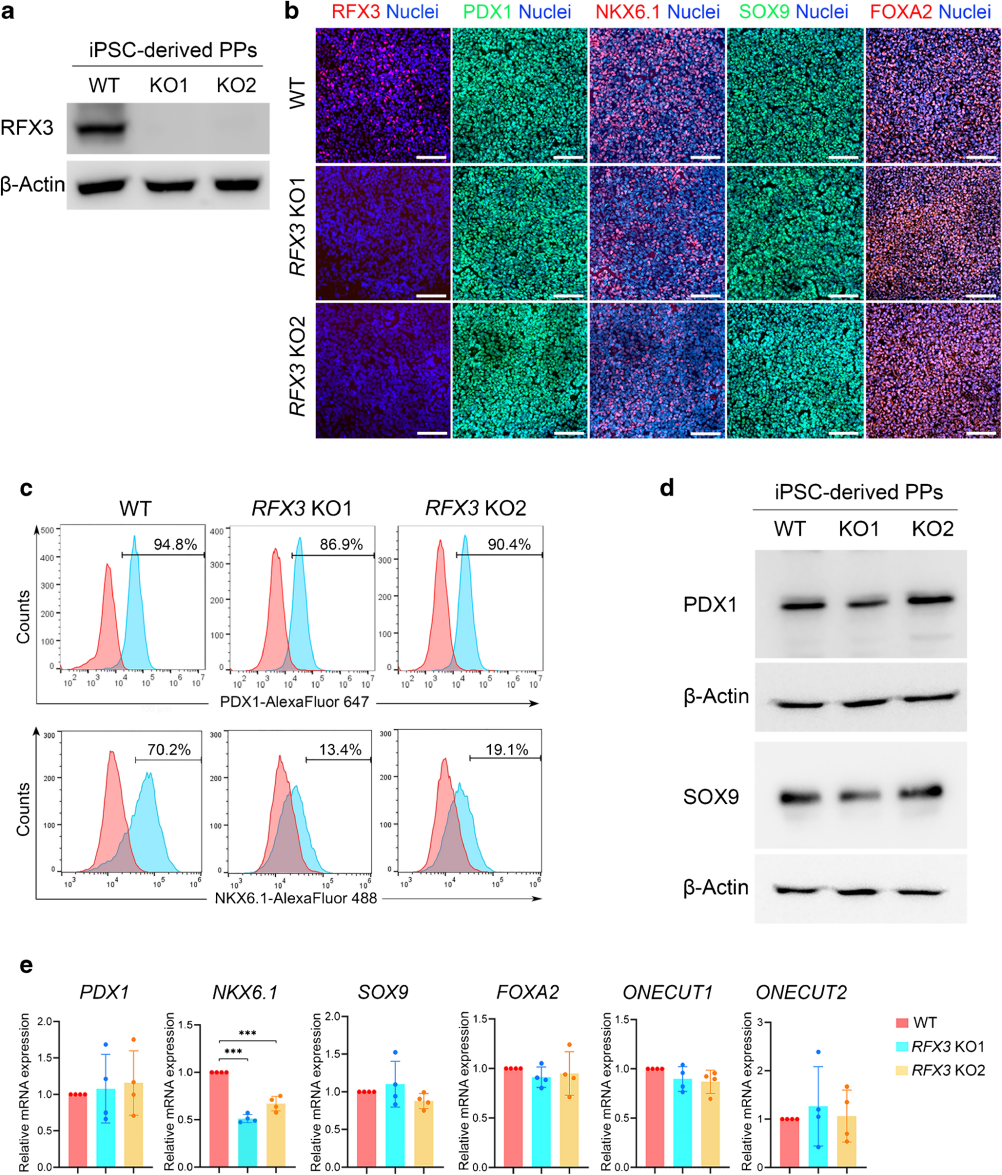
Impact of *RFX3* deletion on iPSC-derived PPs. (**a**) Western blotting analysis validating the loss of RFX3 protein expression in *RFX3* KO PPs. (**b**) Immunofluorescence images showing expression of RFX3, PDX1, NKX6.1, SOX9 and FOXA2 in *RFX3* KO PPs and WT PPs (images representative of at least three biological experiments). (**c**) Representative flow cytometry histograms showing the expression of PDX1 and NKX6.1 in *RFX3* KO PPs and WT PPs. (**d**) Representative western blots showing the expression levels of PDX1 and SOX9 proteins in *RFX3* KO PPs and WT PPs. (**e**) RT-qPCR analysis for PP developmental markers (*n*=4). Relative mRNA expression calculated as fold change vs WT (set as 1). Data are presented as means±SD. ***p<0.001. Scale bar, 100 µm

To investigate the functional role of RFX3 in human islet development, we generated *RFX3* mutant iPSCs by targeting exon 3 to introduce indels using CRISPR/Cas9 in WT iPSCs. The resulting frameshift mutations led to introduction of a premature stop codon in exon 4. Successfully edited clones were, thus, selected following validation using sanger sequencing (ESM Fig. 3a). Furthermore, upon differentiation to the PP stage, the generated *RFX3* KO iPSC lines showed complete absence of RFX3 expression when examined by western blotting and immunofluorescence (Fig. 2a, b). The selected *RFX3* KO iPSCs expressed key pluripotency markers and had normal karyotypes, comparable with those of WT iPSCs (ESM Fig. 3b, c). We then differentiated these *RFX3* KO iPSC lines into SC-islets to evaluate the effect of *RFX3* loss across the PP, EP and islet stages that exhibited RFX3 expression. Our analysis revealed that PPs lacking *RFX3* exhibited no significant change in the protein and mRNA levels of key progenitor markers, such as PDX1, SRY-box transcription factor 9 (SOX9) and forkhead box A2 (FOXA2) (Fig. 2b–e). However, NKX6.1 expression was significantly reduced at protein and mRNA levels (Fig. 2b, c, e). RT-qPCR analysis showed no significant change in the mRNA levels of other progenitor markers, *ONECUT1* and *ONECUT2* (Fig. 2e).

**Fig. 3:**
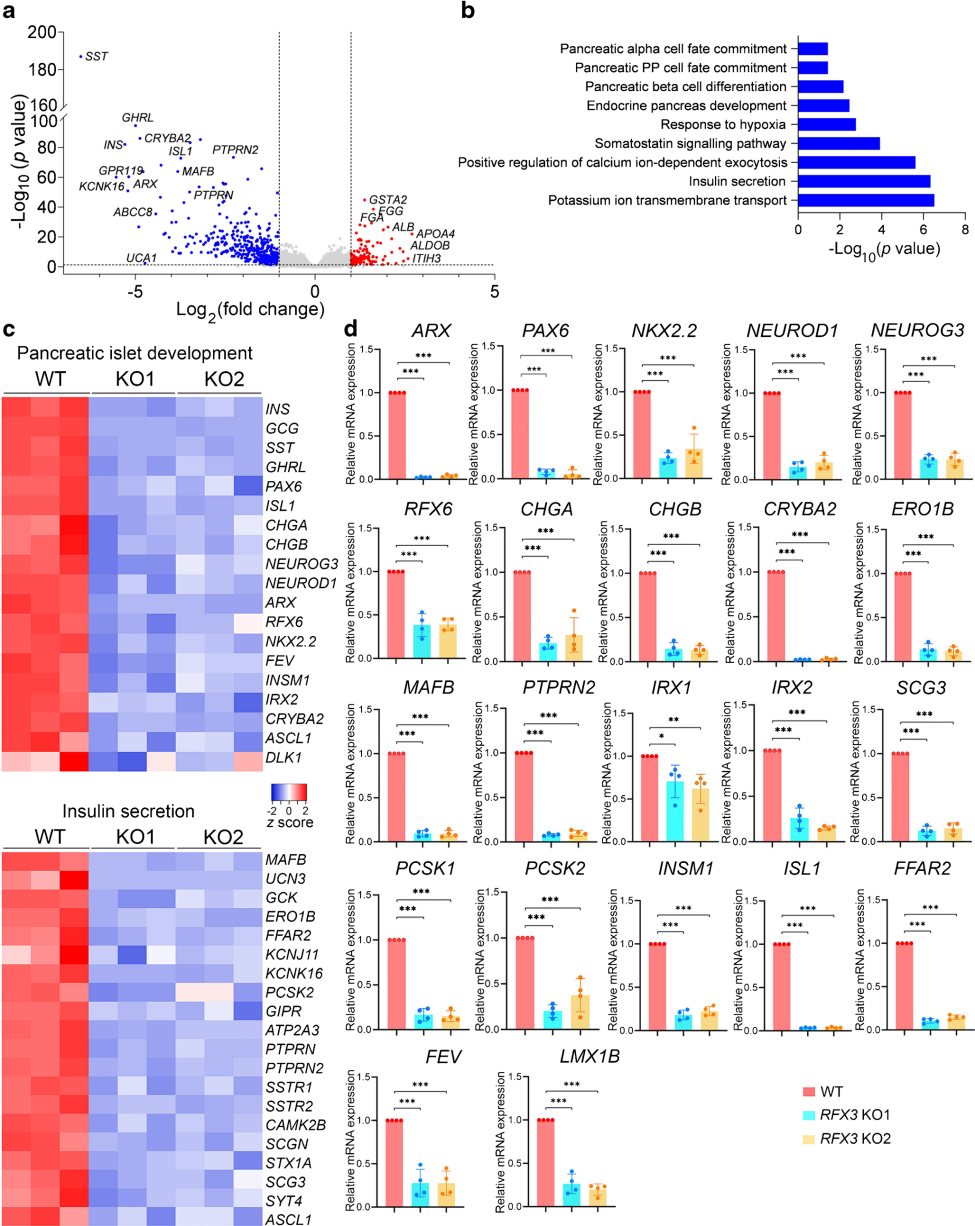
Abolished endocrine specification in iPSC-derived PPs lacking *RFX3*. DEGs and pathways were identified by bulk RNA-seq analysis of *RFX3* KO PPs and WT PPs (*n*=3). (**a**) Volcano plot showing upregulated (red) and downregulated (blue) DEGs. (**b**) GO of significantly enriched biological processes in downregulated DEGs. (**c**) Heatmaps depicting *z* scores of significantly downregulated DEGs regulating pancreatic islet development and insulin secretion. (**d**) RT-qPCR analysis of key DEGs affected by *RFX3* deletion in iPSC-derived PPs (*n*=4). Relative mRNA expression calculated as fold change vs WT (set as 1). Data are presented as means±SD. *p<0.05, **p<0.01, ***p<0.001

To gain further insight into the impact of *RFX3* loss on the pancreatic gene network, we conducted bulk RNA-seq analysis on *RFX3* KO PPs and WT PPs. The transcriptome analysis revealed significant dysregulation in endocrine specification processes in *RFX3* KO PPs. We identified 378 downregulated differentially expressed genes (DEGs) (log_2_[fold change] <−1.0, adjusted *p* value <0.05) and 113 upregulated DEGs (log_2_[fold change] >1.0, adjusted *p* value <0.05) in *RFX3* KO PPs compared with WT PPs (Fig. 3a and ESM Tables 4, 5, ESM Fig. 4a). Gene ontology (GO) analysis of the downregulated biological processes in *RFX3* KO PPs showed significant enrichment related to insulin secretion, potassium and calcium ion transport, pancreatic islet cell and endocrine pancreas development, and response to hypoxia (Fig. 3b and ESM Figs 4b and 5). However, pathways related to cholesterol and triacylglycerol homeostasis, negative regulation of cell apoptotic process, and cellular oxidant detoxification were upregulated in *RFX3* KO PPs, indicating a shift in lineage specification in *RFX3* KO PPs (ESM Fig. 4b). The heatmaps for the selected top significantly downregulated DEGs governing islet development and insulin secretion suggest a disrupted endocrine programme in PPs lacking *RFX3* (Fig. 3c). Furthermore, RT-qPCR validation confirmed that several key transcriptional regulators essential for islet development, such as *ARX*, *PAX6*, *NKX2.2*, *NEUROD1*, *NEUROG3*, *RFX6*, *CHGA*, *CHGB*, *CRYBA2*, *ERO1B, MAFB*, *PTPRN2*, *IRX1*, *IRX2*, *SCG3*,* PCSK1*, *PCSK2*, *INSM1*, *ISL1*, *FFAR2*, *FEV* and *LMX1B*, were significantly downregulated in *RFX3* KO PPs (Fig. 3d). Interestingly, comparison with recent RNA-seq results from *RFX6* KO PPs [8] revealed that only 32.5% of the downregulated genes and 29.2% of the upregulated genes in *RFX3* KO PPs were also downregulated and upregulated, respectively, in *RFX6* KO PPs [8] (ESM Fig. 6a, b). GO analysis of these commonly downregulated DEGs revealed enrichment in biological processes related to insulin secretion, potassium and calcium ion transport, regulation of exocytosis, enteroendocrine cell differentiation, glucose homeostasis and the SST signalling pathway (ESM Fig. 6c). The commonly upregulated DEGs were associated with cholesterol and triglyceride homeostasis, fibrinolysis, lipoprotein metabolism, positive regulation of of phosphoinositide-3-kinase (PI3K)–Akt signalling, and negative regulation of the apoptotic process (ESM Fig. 6d).

### *RFX3* deficiency reduces pancreatic endocrine genes and increases EC genes in iPSC-derived EPs

**Fig. 4:**
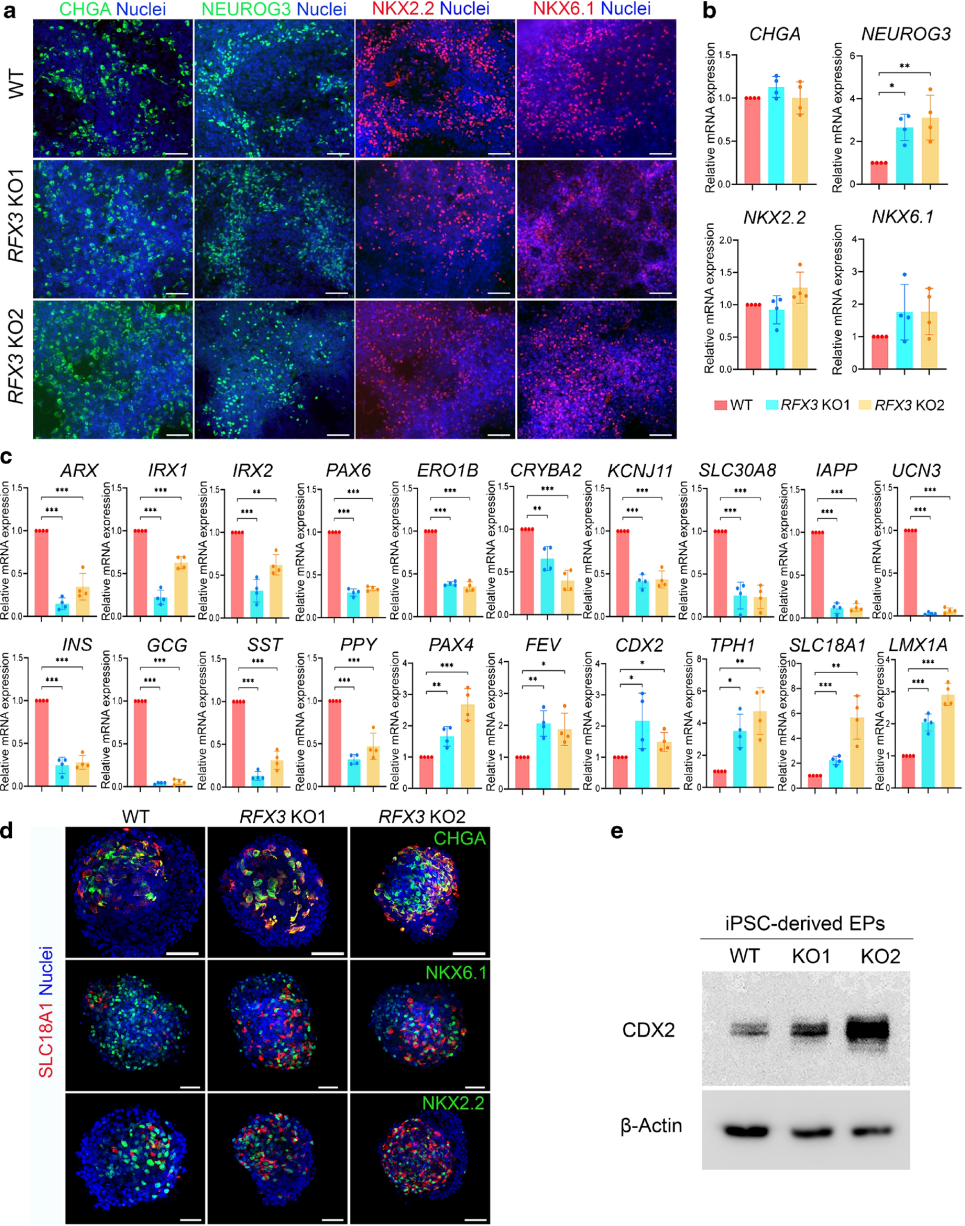
Effect of *RFX3* deletion on EPs derived from iPSCs. (**a**, **b**) Representative immunofluorescence images (*n=*3) (**a**) and RT-qPCR analysis (*n=*4) (**b**) showing the expression of the EP markers CHGA, NEUROG3, NKX2.2 and NKX6.1, in EPs derived from WT and *RFX3* KO iPSCs. (**c**) RT-qPCR analysis showing reduced expression of key markers regulating endocrine pancreas differentiation, along with increased expression of EC markers in EPs derived from *RFX3* KO iPSCs compared with WT iPSCs (*n*=4). Relative mRNA expression calculated as fold change vs WT (set as 1). (**d**) Representative immunofluorescence images showing the co-expression of the EC marker SLC18A1 with CHGA, NKX6.1 and NKX2.2 in *RFX3* KO EPs compared with WT EPs (*n*=3). (**e**) Western blotting analysis showing an increase in the expression levels of CDX2 in *RFX3* KO EPs compared with WT EPs (*n*=3). Data are presented as means±SD. *p<0.05, **p<0.01, ***p<0.001. Scale bar, 100 µm

Next, we investigated the impact of *RFX3* loss on EPs. Immunofluorescence analysis of EPs derived from *RFX3* KO iPSCs showed no differences in the protein levels of chromogranin A (CHGA), NEUROG3, NKX2.2 and NKX6.1 compared with WT EPs (Fig. 4a). RT-qPCR results were comparable, except for *NEUROG3*, which showed a significant increase in mRNA levels in the *RFX3* KO EPs (Fig. 4b). However, several key genes associated with islet development were significantly downregulated in EPs lacking *RFX3*; these included *ARX*, *IRX1*, *IRX2*, *PAX6*, *ERO1B*, *CRYBA2*, *KCNJ11*, *SLC30A8*, *IAPP*, *UCN3*, *INS*, *GCG*, *SST* and *PPY* (Fig. 4c). Of note, scRNA-seq analysis at the EP stage showed high *RFX3* expression in cell clusters expressing high levels of these endocrine genes (Fig. 1d), suggesting their direct regulation by *RFX3*. Interestingly, genes linked to enterochromaffin cell (EC) development, such as *PAX4*, *FEV*, *CDX2*, *TPH1*, *SLC18A1* and *LMX1A*, were significantly upregulated (Fig. 4c). Immunostaining and western blotting analyses confirmed the upregulation of EC markers solute carrier family 18 member A1 (SLC18A1) and caudal type homeobox 2 (CDX2) in *RFX3* KO EPs compared with WT EPs (Fig. 4d, e). SLC18A1 expression was predominantly co-localised with EP markers CHGA, NKX6.1 and NKX2.2, all of which remained unchanged (Fig. 4d). The unaltered expression of these markers at the EP stage is likely due to the increased EC numbers.

### Loss of *RFX3* disrupts the development of iPSC-derived pancreatic islets

**Fig. 5:**
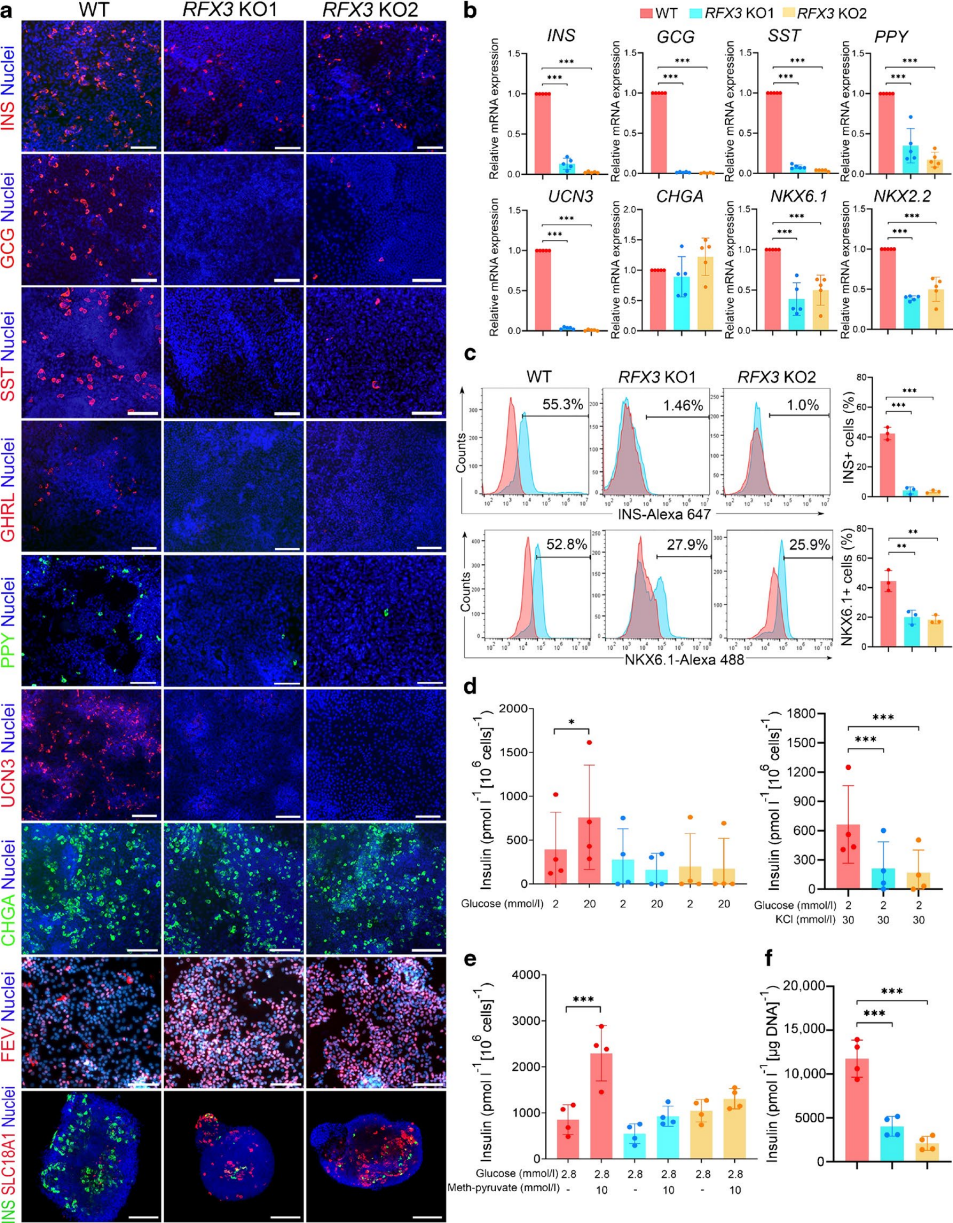
Impaired generation of hormone-secreting islet cells from *RFX3* KO iPSCs. (**a**) Representative immunofluorescence images showing reduced expression of islet hormones (INS, GCG, SST, GHRL and PPY) and UCN3 in islet cells differentiated from *RFX3* KO iPSCs. In contrast, CHGA expression remained unchanged, while FEV and SLC18A1 expression increased (*n*=4). (**b**) RT-qPCR analysis for key islet markers in WT islets and *RFX3* KO islets (*n*=5). (**c**) Flow cytometry analysis and quantification of the expression of INS and NKX6.1 in islets differentiated from *RFX3* KO iPSCs compared with WT controls (*n*=3). (**d**) Glucose-stimulated insulin secretion assay illustrating diminished beta cell functionality and reduced insulin release upon treatment with high glucose (20 mmol/l, *n*=4) and KCl (30 mmol/l, *n*=4), respectively. (**e**) Insulin release in response to 10 mmol/l methyl pyruvate from *RFX3* KO islets compared with WT controls. (**f**) Total insulin content was measured from lysed islet organoids derived from *RFX3* KO iPSCs compared with WT controls using acid-ethanol (*n*=4). Data are presented as means±SD. *p<0.05, **p<0.01, ***p<0.001. Scale bar, 100 µm

To assess the impact of *RFX3* absence on endocrine pancreas formation and functionality, we evaluated SC-islet differentiation using different approaches. Immunofluorescence and RT-qPCR analyses revealed reduced expression of islet hormones including INS, GCG, SST, ghrelin (GHRL), PPY and urocortin 3 (UCN3), while CHGA expression remained unaffected (Fig. 5a, b). Interestingly, FEV and SLC18A1, EC markers, were upregulated in *RFX3* KO islets (Fig. 5a). Flow cytometry quantification showed a decreased percentage of INS^+^ and NKX6.1^+^ cells in *RFX3* KO islets compared with WT islets (Fig. 5c).

To enhance the functional properties of SC-islets, we re-aggregated iPSC-derived PPs into 3D organoids and differentiated them into SC-islets. We then performed glucose-stimulated insulin secretion assays on these SC-islet organoids to explore the impact of *RFX3* deficiency. Upon treatment with high glucose concentrations (20 mmol/l), *RFX3* KO SC-islets showed an abolished insulin secretion response compared with WT organoids (Fig. 5d). Furthermore, stimulation with the depolarising agent KCl failed to induce an adequate insulin secretion response from *RFX*3 KO SC-islets (Fig. 5d). The same finding was observed in response to the mitochondrial fuel methyl pyruvate, where the insulin secretion was significantly reduced in *RFX3* KO islets compared with WT controls (Fig. 5e). In addition, total insulin content measured after lysing the organoids with acid-ethanol was significantly reduced in the *RFX*3 KO SC-islets compared with WT controls (Fig. 5f). This indicates that *RFX3* depletion impairs the functionality of pancreatic beta cells by diminishing their insulin secretion capacity.

**Fig. 6:**
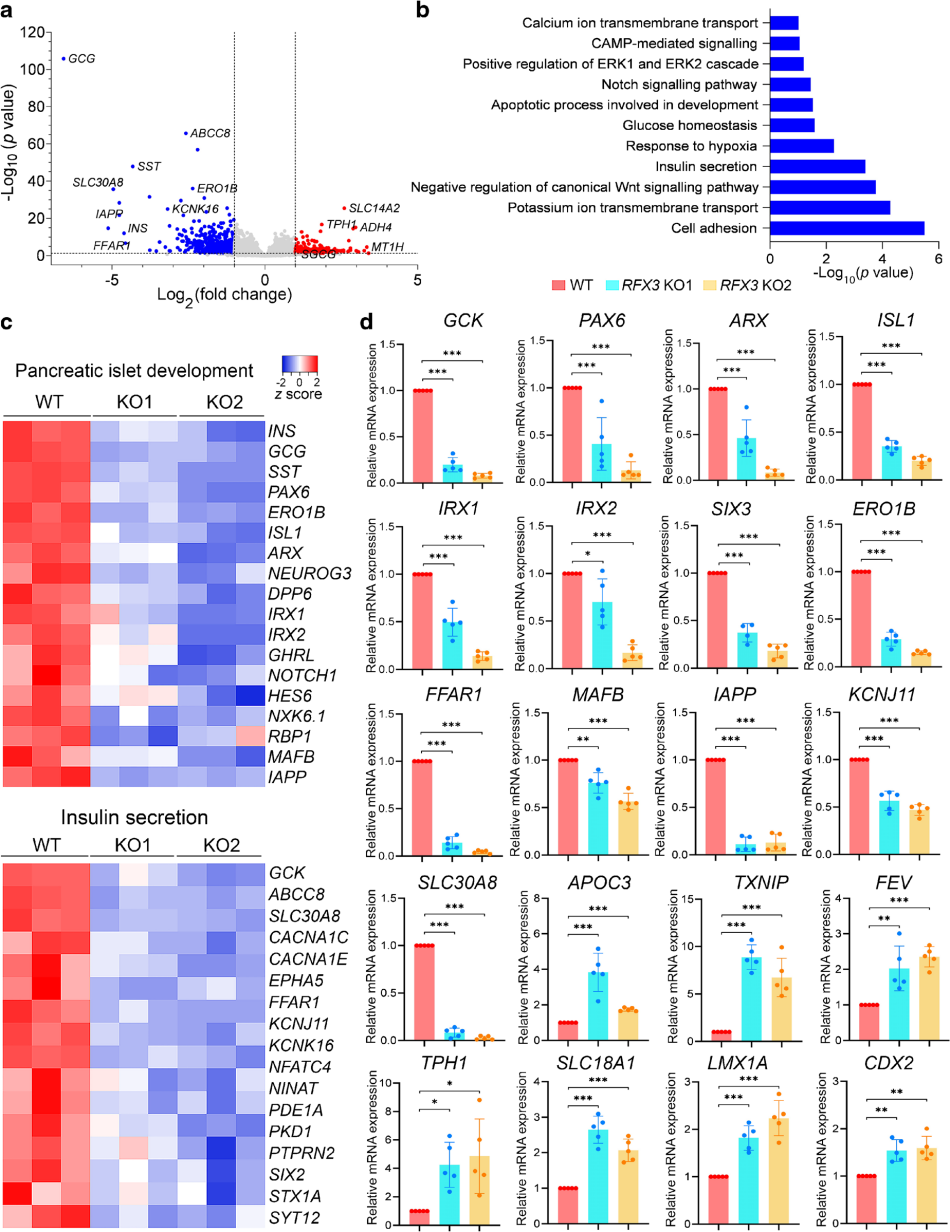
Transcriptome changes in pancreatic islets lacking the *RFX3* gene. (**a**) Volcano plot depicting DEGs identified by transcriptome analysis of islet cells derived from *RFX3* KO iPSCs and WT iPSCs (*n*=3). Significantly upregulated DEGs are shown in red; downregulated DEGs are shown in blue. (**b**) GO of downregulated biological processes in *RFX3* KO islets compared with WT islets. (**c**) Heatmaps for key DEGs involved in pancreatic islet development and insulin secretion depicting their *z* scores. (**d**) RT-qPCR analysis showing downregulation of key islet markers and upregulation of genes associated with ECs in iPSC-derived islets lacking *RFX3* (*n*=5). Relative mRNA expression calculated as fold change vs WT (set as 1). Data are presented as means±SD. *p<0.05, **p<0.01, ***p<0.001

To gain a deeper understanding of the role of RFX3 in SC-islets, we performed RNA-seq analysis on *RFX3* KO islets and WT islets. The transcriptomic data revealed hampered islet development due to the dysregulation of key endocrine genes (Fig. 6a–c and ESM Fig. 4). We identified 494 significantly downregulated DEGs (log_2_[fold change] <−1.0, adjusted *p* value <0.05) and 249 upregulated DEGs (log_2_[fold change] >1.0, adjusted* p* value <0.05) in *RFX3* KO islets compared with WT islets (ESM Tables 6, 7). Islet hormone genes *INS*, *GCG*, *SST*, and *GHRL* were downregulated, consistent with the immunostaining and RT-qPCR results (Fig. 6c, d). Furthermore, genes directly targeted by *RFX3*, such as *GCK* [5], and other markers of pancreatic endocrine differentiation, were also downregulated (Fig. 6a, c, d). Analysis of downregulated DEGs revealed significant enrichment in pathways related to insulin secretion, potassium and calcium ion transport, and response to hypoxia, among others associated with islet development and function (Fig. 6b, c and ESM Fig. 5). However, upregulated DEGs were associated with liver and fat-cell development, proteolysis, triacylglycerol biosynthesis, lipid and glycerol metabolic processes, and urea metabolism (ESM Fig. 4b). RT-qPCR validation confirmed a significant downregulation of pancreatic endocrine genes (*PAX6*, *ARX*, *ISL1*, *IRX1*, *IRX2*, *SIX3*, *ERO1B*, *FFAR1*, *MAFB*, *IAPP*, *KCNJ11* and *SLC30A8*) and upregulation of *APOC3* and *TXNIP* (a pro-apoptotic gene) [18] (Fig. 6d). Interestingly, the EC markers *FEV*, *TPH1*, *SLC18A1*, *LMX1A* and *CDX2* were significantly upregulated in *RFX3* KO islets compared with controls (Fig. 6d). These findings indicate that RFX3 is essential for pancreatic islet formation and suppressing the development of ECs, which are linked to beta cell immaturity [17].

RFX3 is known to play a key role in ciliogenesis [19]. To explore this, we analysed the DEGs associated with cilia function using the CiliaMiner database (https://kaplanlab.shinyapps.io/ciliaminer/). We found that several cilia-regulatory genes were significantly downregulated in PPs and islets lacking *RFX3*, highlighting the impact of *RFX3* on cilia function (ESM Fig. 7).

### *RFX3* deficiency reduces the viability of iPSC-derived islet organoids

**Fig. 7:**
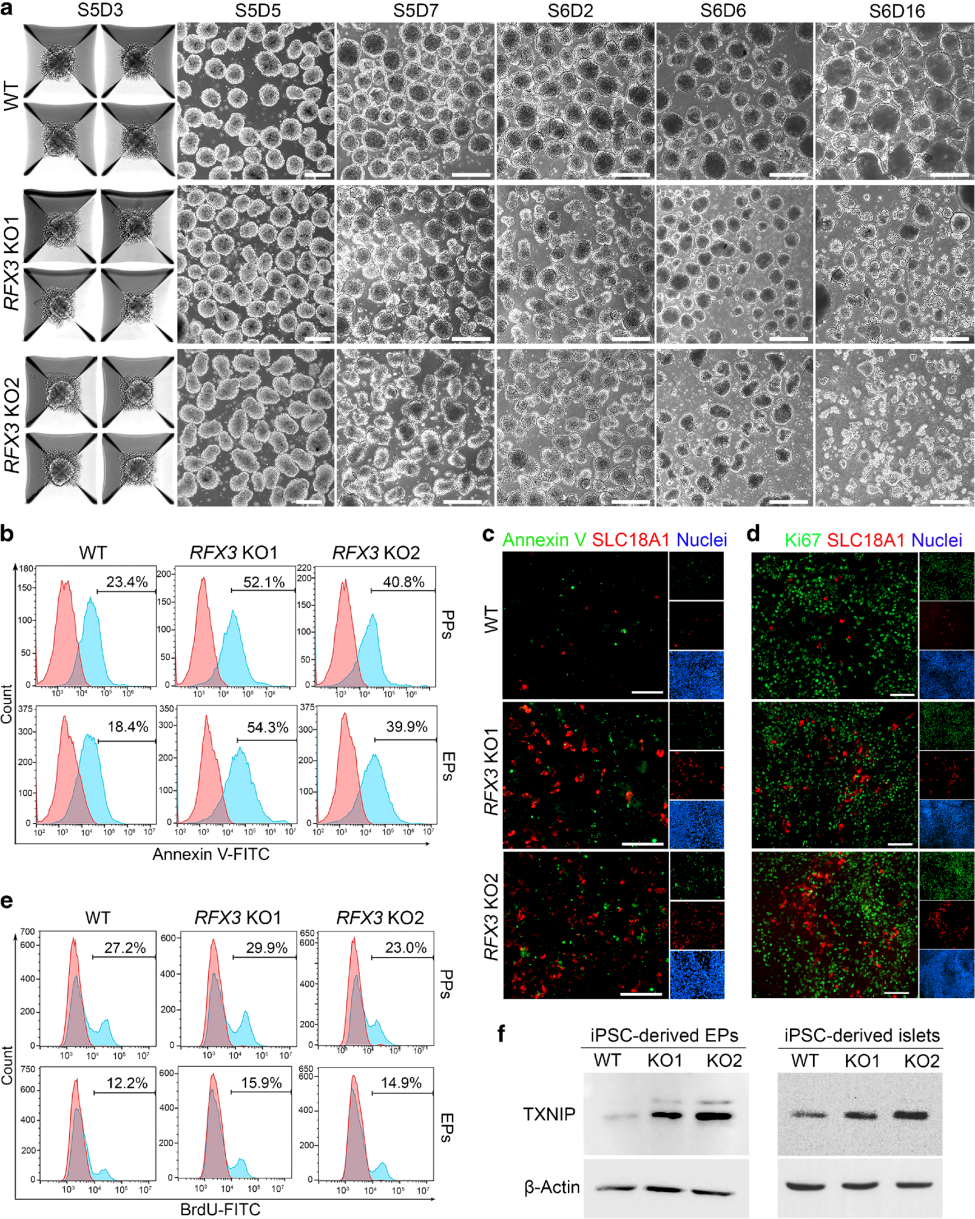
*RFX3* loss leads to increased apoptosis and disruption of iPSC-derived islet organoids. (**a**) Representative phase contrast images showing morphological changes of islet organoids during stages 5 and 6 (EPs and islets, respectively) derived from *RFX3* KO iPSCs and WT iPSCs (*n*=3). Note the reduced size of organoids derived from *RFX3* KO iPSCs compared with WT controls. ‘S’ denotes stage; ‘D’ denotes day. (**b**) Flow cytometry quantification of apoptotic (Annexin V^+^) cells in stages 4 (PPs) and 5 (EPs) indicating increased apoptosis in *RFX3*-deficient cells. (**c**) Immunostaining images showing an increase in the number of cells expressing the apoptosis marker Annexin V and the EC marker SLC18A1 in *RFX3*-deficient EPs, with no co-localisation observed between the two markers (*n*=2). (**d**) Immunostaining images showing no change in the expression of the proliferation marker Ki67 and an increase in the expression of SLC18A1 in *RFX3*-deficient EPs, with no co-localisation observed between the two markers (*n*=2). (**e**) Flow cytometry analysis of BrdU incorporation showing no significant changes in cell proliferation (BrdU^+^ cells) in PPs and EPs derived from *RFX3* KO iPSC lines compared with those from WT iPSCs. (**f**) Western blotting analysis showing an increase in the expression levels of TXNIP in EPs and islets derived from *RFX3* KO iPSCs compared with WT controls (representative of *n*=3). Scale bars, 100 µm in (**a**) and 200 µm in (**c**, **d**)

During SC-islet organoid differentiation, we observed dramatic morphological differences between *RFX3* KO and WT organoids (Fig. 7a). In *RFX3* KO organoids, the structure deteriorated into smaller clumps of dissociating cells during stage 5 (EP), and this abnormal morphology persisted into stage 6 (islets), unlike the stable morphology in WT organoids (Fig. 7a). To determine whether the reduced organoid size is due to increased apoptosis or decreased cell proliferation, we assessed both processes. Flow cytometry revealed a significant increase in Annexin V^+^ (apoptotic) cells in pancreatic cells derived from *RFX3* KO iPSCs at both PP and EP stages (Fig. 7b). Annexin V did not co-localise with the EC marker SLC18A1 in EPs (Fig. 7c), suggesting that the increased EC number in *RFX3* KO EPs is not linked to cell death. Examination of SLC18A1 and the proliferation marker Ki67 confirmed an increase in the EC marker, with no change in Ki67 expression and no co-localisation between the two markers (Fig. 7d). Quantification of BrdU incorporation further confirmed no difference in proliferation rates between WT and KO cells (Fig. 7e). To explore the mechanism of cell death, we analysed thioredoxin-interacting protein (TXNIP) level, which was upregulated in our RNA-seq and RT-qPCR analyses. Elevated TXNIP is known to induce pancreatic beta cell apoptosis [18]. Western blotting confirmed elevated TXNIP levels in EPs and SC-islets lacking *RFX3* compared with WT controls (Fig. 7f). These results suggest that RFX3 loss impairs SC-islet viability during development by increasing TNXIP levels.

### RFX3 overexpression reverses the expression of dysregulated genes in PPs and EPs lacking RFX3

Aiming to rescue the impact of *RFX3* loss on iPSC-derived pancreatic cells, we induced RFX3 overexpression (RFX3 OE) on day 4 of stage 4 (PPs) and assessed its effect on PPs and EPs (ESM Fig. 8). At the PP stage, 48 h post-transfection, RFX3 OE significantly increased the mRNA expressions of endocrine markers that were downregulated in *RFX3* KO PPs, including *RFX3*, *NKX6.1*, *ARX*, *PAX6*, *NKX2.2*, *NEUROD1*, *NEUROG3*, *CHGA*, *CHGB*, *CRYBA2*, *ERO1B*, *MAFB*, *PTPRN2*, *IRX1*, *IRX2*, *SCG3*, *PCSK1*, *PCSK2*, *INSM1*, *ISL1*, *FFAR2*, *FEV* and *LMX1B* (ESM Fig. 8a). Next, we tested the impact of RFX3 OE on dysregulated DEGs on day 3 of stage 5 (EPs) and found that there was a significant increase in the expression of *PAX6*, *ERO1B*, *CRYBA2*, *KCNJ11*, *SLC30A8*, *IAPP*, *UCN3*, *INS*, *GCG*, *SST* and *PPY* (ESM Fig. 8b). In contrast, RFX3 OE resulted in the downregulation of significantly upregulated markers, such as *NEUROG3* and *PAX4*, as well as EC markers *FEV*, *CDX2*, *TPH1*, *SLC18A1* and *LMX1A*, in *RFX3* KO EPs (ESM Fig. 8b).

## Discussion

Rodent studies have demonstrated that RFX3 is important for pancreatic islet cell development [4, 5]. Recent scRNA-seq studies of SC-islets further highlighted *RFX3* expression in multiple pancreatic endocrine cell populations, particularly during later stages of islet development [17, 20, 21]. However, its role in human islet development remains unexplored. Using SC-islet organoids, we modelled *RFX3* loss-of-function and found that RFX3 expression began in PPs and persisted in endocrine cell populations throughout differentiation. Loss of *RFX3* significantly reduced the expression of genes critical for endocrine islet development, led to smaller SC-islet organoids due to increased cell death, and impaired islet cell development. Furthermore, RFX3 deficiency resulted in increased expression of EC-specific genes.

Our findings partially align with those of rodent studies, where *Rfx3*^−/−^ mice and those with pancreas-specific *Rfx3* deletion fail to develop INS-, GCG- and SST-secreting cells and show decreased glucokinase expression [4, 5]. However, in human SC-islets, RFX3 deficiency led to a reduced number of PPY^+^ endocrine cells and significant cell death during endocrine specification, resulting in smaller organoids, unlike rodent models that showed no impact on cell survival or proliferation [5]. Our human iPSC model revealed decreased expression of key endocrine regulators during differentiation. Single-cell analysis showed that RFX3 was associated with endocrine clusters, with the highest expression occurring in specific subpopulations at different stages, such as *CHGA*^high^ *NEUROD1*^high^, *POU2F2*^high^ *PAX4*^high^ and *POU2F2*^high^ *RFX3*^high^ in PPs, EPs and islets, respectively. These clusters also included key pancreatic endocrine regulators such as *CHGA*, *FEV*, *NEUROD1*, *NKX2.2*, *NEUROG3*, *RFX6* and others, supporting RFX3’s role in pancreatic endocrine specification*.* As in rodent models [5], RFX3 is not involved in exocrine pancreas development, as it was absent from exocrine gene-expressing clusters. In SC-islets, *RFX3* was prominently expressed in clusters representing alpha, beta, delta, and EC cells, underscoring its role in regulating pancreatic endocrine cell function and identity.

In this study, we observed that while *RFX3* loss significantly reduced islet hormones, the pan-endocrine marker CHGA remained unchanged at both the EP and islet stages. This may be due to an increase in ECs, which also express CHGA [13]. A recent study identified three types of CHGA^+^ cells in EPs and islets derived from hPSCs: beta, alpha and ECs [13]. Although ECs are present in fetal and hPSC-derived islets [13, 17, 20, 22], they decline with culture and are absent in the adult pancreas [17, 20]. Our results showed elevated EC marker expression in *RFX3*-deficient EP and islet cells, suggesting that increased EC number accounts for the unchanged CHGA expression despite fewer islet cells. We also noticed increased *NEUROG3* and *PAX4* expression, with unchanged NKX6.1 and NKX2.2 levels, in *RFX3* KO EPs. Furthermore, the EC marker SLC18A1 was upregulated in *RFX3* KO EPs and co-localised with CHGA, NKX6.1 and NKX2.2, confirming their expression in ECs. NKX6.1 binds LIM homeobox transcription factor 1α (LMX1A), a transcription factor for serotonin (5HT) synthesis [17, 23], and is reduced in pancreatic endocrine cells lacking *CDX2*, a key regulator of EC genes such as tryptophan hydroxylase 1 (TPH1) [24] and SLC18A1 [17]. hPSC-derived ECs represent a transient, beta cell-related population that decreases with maturation, as indicated by the reduced percentage of NKX6.1^+^ SLC18A1^+^ hPSC-ECs [17]. In human fetal [25] and SC-islets, the 5HT-producing pre-beta cell population expresses high levels of CHGA, SLC18A1, LMX1A, FEV, paired box 4 (PAX4), NEUROG3 and CDX2 [13, 17, 20, 21]. Our results showed significant upregulation of these EC markers in *RFX3*-deficient EPs and islets, indicating that RFX3 is crucial for islet development by promoting endocrine specification and inhibiting EC differentiation.

We observed that 32.5% of downregulated genes in *RFX3* KO PPs were also downregulated in *RFX6* KO PPs [8], primarily related to endocrine specification. Furthermore, 67.46% were uniquely downregulated in *RFX3* KO PPs, indicating that while RFX3 and RFX6 share some phenotypes, they have distinct downstream mechanisms. *RFX3* deficiency increased expression of EC markers, while *RFX6* loss reduced them in our study [8]; however, another study found that *RFX6* loss increased EC markers [7]. Both cause apoptosis and smaller islet organoids, albeit through different mechanisms (*RFX6* loss reduces catalase [8], while catalase remains unaffected in *RFX3*-deficient cells). Instead, *RFX3* loss increased TXNIP, a pro-apoptotic protein linked to diabetes and oxidative stress-induced beta cell death [26–28]. These findings suggest that *RFX3* loss impairs islet survival by elevating TXNIP levels and disrupting endocrine specification.

While *RFX6* mutations are linked to neonatal diabetes and type 2 diabetes [29–33], the role of RFX3 in diabetes is less understood. A recent report identified RFX3 as part of an islet-specific enhancer complex, interacting with key genes such as *GLIS3* [34]. Deletion of this complex reduces RFX3 expression, underscoring its importance in islet function and type 2 diabetes susceptibility [34]. The relationship between RFX3 and genes involved in monogenic diabetes has been established. Key transcription factors such as PDX1, one cut homeobox 1 (ONECUT1) and NEUROG3, which are crucial for islet development, bind to the RFX3 regulatory region. ChIP-seq analysis in iPSC-derived PPs has shown that PDX1directly activates the RFX3 promoter [35]. Similarly, ONECUT1 and NEUROG3 regulate RFX3 expression in hESC-derived PPs and iPSC-derived EPs, respectively [36, 37]. These findings highlight the critical role of RFX3 in islet function and its possible impact on diabetes risk, although further studies are needed to understand its role in diabetes.

In conclusion, our study highlights the essential role of RFX3 in human pancreatic islet development. We found that RFX3 is highly expressed in endocrine cells throughout SC-islet development. Its absence impairs the formation of all pancreatic hormone-secreting cells, primarily due to decreased expression of key endocrine genes and increased cell apoptosis, associated with elevated TXNIP levels. Notably, RFX3 loss leads to increased EC cell formation, underscoring its critical role in beta cell maturation. These results contrast with rodent studies, which do not report similar changes in apoptosis or beta cell and EC specification. Such differences may arise from technological advances or physiological differences between human and rodent islets. Our findings establish RFX3 as a crucial regulator necessary for the proper formation and maturation of human islet cells.

## Supplementary Information

Below is the link to the electronic supplementary material.

ESM (PDF 2452 KB)

## Data Availability

The RNA-seq datasets have been submitted to the Zenodo repository and can be accessed via the following links: DOI 10.5281/zenodo.13647651 (PPs); and DOI 10.5281/zenodo.13762055 (SC-islets).

## Abbreviations

CDX2: Caudal type homeobox 2
CHGA: Chromogranin A
DEG: Differentially expressed gene
EC: Enterochromaffin cell
EP: Endocrine progenitor
FOXA2: Forkhead box A2
GCG: Glucagon
GHRL: Ghrelin
GO: Gene Ontology
hESC: Human embryonic stem cell
hPSC: Human pluripotent stem cell
5HT: Serotonin
INS: Insulin
iPSC: Induced pluripotent stem cell
KO: Knockout
LMX1A: LIM homeobox transcription factor 1α
NEUROG3: Neurogenin 3
NKX2.2: NK2 homeobox 2
NKX6.1: NK6 homeobox 1
ONECUT1: One cut homeobox 1
PAX4: Paired box 4
PDX1: Pancreatic and duodenal homeobox 1
PP: Pancreatic progenitor
PPY: Pancreatic polypeptide Y
RFX3: Regulatory factor X3
RFX6: Regulatory factor X6
RT-qPCR: Real-time quantitative PCR
SC-islets: IPSC-derived islets
SLC18A1: Solute carrier family 18 member A1
SOX9: SRY-box transcription factor 9
SST: Somatostatin
TXNIP: Thioredoxin-interacting protein
UCN3: Urocortin 3
WT: Wild-type
