# Supplementary material for: RFX3 is essential for the generation of functional human pancreatic islets from stem cells
Source: Diabetologia. 2025 Apr 23;68(7):1476–91. doi: 10.1007/s00125-025-06424-4 (PMC12176927; doi:10.1007/s00125-025-06424-4)
Supplement: Supplementary file 1 — ESM (PDF 2452 KB) [file 125_2025_6424_MOESM1_ESM.pdf]

Electronic Supplementary Material (ESM)

RFX3 is essential for the generation of functional human pancreatic islets from stem cells

Bushra Memon[#], Noura Aldous[#], Ahmed K. Elsayed[#], Sadaf Ijaz, Sikander Hayat, Essam M. Abdelalim*

# *These authors equally contributed to this work*

***Corresponding author**

Essam M. Abdelalim, PhD

E-mail: emohamed3@sidra.org

This ESM includes:

- ESM Methods
- ESM Tables 1 to 7
- ESM Figures 1 to 8

ESM Methods

hPSC maintenance and *RFX3* KO iPSC generation

iPSCs were generated in our lab from healthy individual, and they were fully characterized and maintained in culture as described in our previous report [1]. iPSCs, hESC-H9, and HA-RFX6 tagged H9 hESCs (RFX6$^{HA/HA}$ H9-hESCs) were cultured on 1:80 diluted geltrex in Knockout DMEM media (Gibco, Massachusetts, USA), and cultured using mTeSR Plus medium (Stem Cell Technologies, Canada) supplemented with 1% Penicillin-streptomycin (ThermoFisher Scientific, Massachusetts, USA).

A CRISPR/Cas9 technique was used to generate *RFX3* KO iPSC lines. Guide RNA (gRNA) targeting *RFX3* coding region was cloned into a GFP-tagged vector expressing spCas9 and transfected into undifferentiated iPSCs, dissociated into single cells, using Lipofectamine 3000 reagent (ThermoFisher Scientific, Massachusetts, USA) using manufacturer's protocol. The transfected iPSCs were then plated on 1:80 geltrex for 48 hours prior to sorting GFP-expressing iPSCs. The isolated single cells were then cultured and expanded as individual clones. DNA was extracted using QuickExtract DNA reagent (Invitrogen, Massachusetts, USA) and the target region was amplified using primers flanking the gRNA binding site. PCR products were used for sanger sequencing for KO verification. Four *RFX3* KO clones were generated and loss of RFX3 protein expression was confirmed using immunofluorescence upon differentiating to pancreatic progenitors (PPs). Finally, two *RFX3* KO clones were selected for further experiments (*RFX3* KO1 and *RFX3* KO2 iPSCs).

Differentiation of hPSCs into islet organoids

Differentiation was started when iPSCs plated on 1:50 geltrex reached 70-80% confluency. Differentiation was performed in adherent culture until the PPs using our o ptimized protocol [2]. Further differentiation to beta cells was performed either in 2D format or 3D where PPs were dissociated into single cells and 2 x 10^6 cells of WT and *RFX3* KO PPs were re-aggregated in Aggrewell 400 plates for organoid formation using PP differentiation media supplemented with 10 µM ROCKi and extended for 48 hours in PP media. After 48 hours, the organoids were transferred onto 6-well ultra-low attachment plates in EP Day 1 differentiation media (ESM Fig. 1a), adapted from Veres et al. protocol for further differentiation into pancreatic islet cells [3]. Different assays to evaluate islet organoids were performed during the time frame between 7 -14 days of stage 6. Media composition for each of differentiation is provided in ESM Table 1.

Real-time quantitative polymerase chain reaction (RT-qPCR)

Differentiated cells were treated with TRIzol reagent (Life Technologies, California, USA) prior to phase separation using chloroform. RNA extraction was performed on the aqueous phase using Direct-zol RNA Miniprep kit (Zymo Research Corporation, California, USA). 1 µg of total RNA was used for cDNA synthesis using High-Capacity cDNA Reverse Transcription Kit while following manufacturer's protocol (Applied Biosystems, Massachusetts, USA). RT-qPCR was performed for the synthesized cDNA using GoTaq qPCR SYBR Green Master Mix (Promega, Wisconsin, USA) for specific primers provided in ESM Table 2. GAPDH was used as an endogenous or housekeeping control while fold changes for KO samples were calculated using the ddCt method taking WT levels as reference.

Western blotting

Total protein was extracted from 1-2 wells of a 6-well plate using RIPA lysis buffer with protease inhibitor (ThermoFisher Scientific, Massachusetts, USA), and protein concentration was measured with the Pierce BCA kit (ThermoFisher Scientific, #23225, Massachusetts, USA). 20 µg of protein were loaded onto 7.5-10% SDS-PAGE gels, separated, and transferred to PVDF membranes (ThermoFisher Scientific, #88518, Massachusetts, USA). Membranes were blocked with 10% skim milk in TBST (tris buffered saline with tween with added 0.5% Tween 20) for at least 2 hours at room temperature. Primary antibodies were diluted in 5% skim milk in TBST and incubated overnight at 4°C, followed by TBST washes. Secondary antibodies were diluted in 5% skim milk in TBST and applied for 1 hour at room temperature, and membranes were washed with TBST for three times. Detection was performed using SuperSignal West Pico Chemiluminescent substrate (ThermoFisher Scientific, #34580, Massachusetts, USA). Antibody details are provided in ESM Table 3.

Immunostaining

Cells were fixed at different timepoints using 4% paraformaldehyde (PFA, Santa Cruz Biotechnology) in phosphate buffered saline (PBS) for 20 min at room temperature followed by permeabilization using PBST (0.5% Triton X-100 in PBS) for 15 mins. Cells were then blocked overnight at 4° C using 6% BSA (bovine serum albumin) in PBST with gentle shaking. For staining, these cells were incubated with primary antibodies overnight at 4° C in 3% BSA in PBST. Washes were performed with TBST. Secondary antibodies were diluted in PBST and added at room temperature for 1 hour and nuclear staining was done using Hoechst 33258 diluted 1:5000 in PBS (Life Technologies, California, USA). Cells were then imaged using inverted fluorescence

microscope (Olympus, Tokyo, Japan). Details for antibodies used in this study are listed in ESM Table 3.

For whole-mount immunostaining, pancreatic organoids at different stages were washed with PBS and fixed in 4% PFA in PBS for 30-40 minutes at room temperature, followed by two washes with TBST. Permeabilization was carried out with 1% PBST (1% Triton X-100 in PBS) for 2-3 hours at room temperature. Whole-mount immunostaining was performed in 1.5 mL Eppendorf tubes, with gentle agitation using a tilt shaker during incubation and washing steps. After permeabilization, clusters were blocked with 6% BSA in 1% PBST overnight and incubated with primary antibodies diluted in 3% BSA in 1% PBST for 48-72 hours at 4°C. Organoids were then washed three times with TBST and incubated with the appropriate Alexa Fluor secondary antibodies in 1% PBST for 2 hours at room temperature. Afterward, clusters were washed three times with TBST and stained with Hoechst 33258 diluted 1:5000 in PBS for 30 minutes, followed by two washes with TBST. The stained clusters were attached to slides, mounted with tissue clearing mounting medium, covered with cover slips, and protected from light until imaging. Imaging was performed using LSM 780 Confocal Microscope (Zeiss, Oberkochen, Germany). Details of the antibodies used in this study are provided in ESM Table 3.

Flow cytometry

Differentiated cells were collected at different stages from one well of a 6-well plate. Cells were dissociated into single cells using TrypLE (ThermoFisher Scientific, Massachusetts, USA), washed with 1 mL of PBS and fixed with cold 4% PFA in PBS for 15 mins at room temperature with gentle shaking. All cell centrifugations were done at 800 ×g for 5 mins and washes were

performed using TBST. Cells were permeabilized using PBST for 20 mins at room temperature and then blocked using 6% BSA in PBS containing 0.1% saponin. Blocking was done overnight at 4° C. Incubation with primary antibodies using 3% BSA in PBS containing 0.1% saponin was done for 3 hours on a shaker, at room temperature. Cells were washed with TBST then incubated with secondary antibodies diluted in 3% BSA in PBS containing 0.1% saponin for 30 minutes at room temperature. Finally, cells were resuspended in PBS, run using BD Accuri C6 Flow Cytometer (BD Biosciences, USA) and analysed using FlowJo software. Antibodies used are listed in ESM Table 3.

Apoptosis and proliferation assays

Differentiated cells at different timepoints were dissociated using TryplE (ThermoFisher Scientific, Massachusetts, USA) and were either used for apoptosis assay or fixed for proliferation assay as previously described [4]. Live dissociated cells were stained with Annexin V using Annexin V-FITC Apoptosis Detection Kit (Abcam, #ab14085) and/or 7-AAD dye (Invitrogen, Massachusetts, USA) as per manufacturer's instructions for 5 mins at 4°C. Samples were analyzed using BD Accuri C6 Flow Cytometer and processed with FlowJo software.

For annexin V cell staining, adherent live cells were washed with PBS then incubated with Annexin V diluted in binding buffer as per manufacturer protocol (Abcam, ab14085) for 30 min at room temperature. Cells were then fixed with 4% PFA in PBS supplemented with 25 mmol/l $CaCl_2$ for 20 min at room temperature. Following steps are similar to that of immunofluorescence while supplementing all buffers with 25 mmol/l $CaCl_2$ to maintain Annexin V stability on fixed cells.

For proliferation assay, the cells were treated with 20 μM BrdU reagent (Invitrogen, Massachusetts, USA) for 5 hours, prior to dissociation and fixation. Dissociated single cells were then fixed with cold 70% ethanol overnight at 4°C. Cells were then rinsed once with PBS and denatured with 2 M HCL containing 0.5% Triton for 15 mins at room temperature, followed by neutralization with 0.1 M sodium tetraborate treatment for 20 mins. Finally, cells were washed with PBS and blocked using 6% BSA in PBS with 0.1% saponin. Cells were then incubated with Alexa Fluor 488-conjugated BrdU monoclonal antibody (B35130, Thermo Fisher, Massachusetts, USA), diluted to 1:100, at room temperature for 2 hours, washed with TBST, and $BrdU^+$ cells were assessed using BD Accuri C6 Flow Cytometer and processed with FlowJo software.

Single cell-RNA sequencing analysis

The online published GSE202497 data set for human pluripotent stem cell differentiation into pancreatic islets (https://www.ncbi.nlm.nih.gov/geo/query/acc.cgi?acc=GSE202497) was used [5] and re-analyzed as previously discussed [6] for time points D11, D14, D21, and D39. As described before, we used top 2000 highly variable genes that were obtained using Seurat V3 algorithm implement in Scanpy from a total of 25,686 cells [6]. Cells from the single-cell data were re-clustered using leiden algorithm after performing technical batch-effect correction using Harmony. Unsupervised clustering was based on the neighborhood graph computed using top 50 batch-effect adjusted principal components. Marker genes for each cluster were calculated using the Wilcoxon method implemented in rank_genes_groups function. Finally, the cell clusters were manually annotated based on expression level for marker genes and are presented in Fig. 1.

Bulk RNA sequencing

RNA sequencing (RNA-seq) results were analyzed as previously described [6]. 1 μg of total RNA was used to purify mRNA using NEBNext Poly(A) mRNA Magnetic Isolation Kit (E7490, New England Biolabs, Massachusetts, USA). RNA-seq libraries were generated using the NEBNext Ultra Directional RNA Library Prep Kit (E7420L, New England Biolabs, Massachusetts, USA), followed by sequencing on an Illumina Hiseq 4000 system. FASTQ files were generated using Illumina BCL2Fastq Conversion Software v2.20. Pair-end FASTQ files were preprocessed with nf-core/rnaseq (version 2.7.2) pipeline, STAR (version 2.7.9a) was used for the read alignments, Salmon (version 1.5.0) for quantification of reads, TrimGalore (version 0.6.6) was applied for read trimming and GENCODE (version 38) was used for annotation of genes. Count matrix was filtered to exclude mitochondrial, ribosomal genes and low expression values were excluded using the HTSFilter (version 1.32.0) [7, 8]. Differentially expressed genes (DEGs) were identified using DESeq2 (version 1.32.0) defined by log2 fold change (FC) > 1 and <-1, with adjusted *p value* < 0.05 and pathway analysis was done using the Database for Annotation, Visualization, and Integrated Discovery (DAVID) [9].

RFX3 overexpression

Single cells were transfected with either RFX3 plasmid (RFX3 (Myc-DDK-tagged), RC201137, OriGene, USA) or an empty pCMV6 vector using Lipofectamine 3000 according to the manufacturer's protocol (ThermoFisher Scientific, #L3000-015, Massachusetts, USA). For stage 4, cells were transfected at the end of day 4 of stage 4 and collected 48 hours later (day 6 of stage 4). For stage 5, cells were transfected at the end of stage 4 and collected 120 hours later (2 days in stage 4 and 3 days in stage 5).

References

[1] Memon B, Elsayed AK, Bettahi I, et al. (2022) iPSCs derived from insulin resistant offspring of type 2 diabetic patients show increased oxidative stress and lactate secretion. Stem Cell Res Ther 13(1): 428. 10.1186/s13287-022-03123-4
[2] Memon B, Karam M, Al-Khawaga S, Abdelalim EM (2018) Enhanced differentiation of human pluripotent stem cells into pancreatic progenitors co-expressing PDX1 and NKX6.1. Stem Cell Res Ther 9(1): 15. 10.1186/s13287-017-0759-z
[3] Veres A, Faust AL, Bushnell HL, et al. (2019) Charting cellular identity during human in vitro beta-cell differentiation. Nature 569(7756): 368-373. 10.1038/s41586-019-1168-5
[4] Aghadi M, Elgendy R, Abdelalim EM (2022) Loss of FOXA2 induces ER stress and hepatic steatosis and alters developmental gene expression in human iPSC-derived hepatocytes. Cell Death Dis 13(8): 713. 10.1038/s41419-022-05158-0
[5] Zhu H, Wang G, Nguyen-Ngoc KV, et al. (2023) Understanding cell fate acquisition in stem-cell-derived pancreatic islets using single-cell multiome-inferred regulomes. Dev Cell 58(9): 727-743 e711. 10.1016/j.devcel.2023.03.011
[6] Aldous N, Elsayed AK, Memon B, Ijaz S, Hayat S, Abdelalim EM (2024) Deletion of RFX6 impairs iPSC-derived islet organoid development and survival, with no impact on PDX1(+)/NKX6.1(+) progenitors. Diabetologia. 10.1007/s00125-024-06232-2
[7] Patro R, Duggal G, Love MI, Irizarry RA, Kingsford C (2017) Salmon provides fast and bias-aware quantification of transcript expression. Nat Methods 14(4): 417-419. 10.1038/nmeth.4197
[8] Rau A, Gallopin M, Celeux G, Jaffrezic F (2013) Data-based filtering for replicated high-throughput transcriptome sequencing experiments. Bioinformatics 29(17): 2146-2152. 10.1093/bioinformatics/btt350
[9] Huang da W, Sherman BT, Lempicki RA (2009) Systematic and integrative analysis of large gene lists using DAVID bioinformatics resources. Nat Protoc 4(1): 44-57. 10.1038/nprot.2008.211

ESM Tables

ESM Table 1. Media formulation and details of cytokine added for *in vitro* differentiation protocol.

Differentiation stage	Media supplement	Cytokines	Total number of days
Stage 1 (DE)	**MCDB 131**:<br>1% Pen/Strep<br>1% L-Glutamine<br>0.5% Fatty acid free BSA<br>1.5 g/l $NaHCO_3$<br>10 mmol/l D-Glucose	**Day 1**:<br>1 µM Y-27632<br>2 µM CHIR99021<br>**Days 1-3**:<br>100 ng/ml Activin A<br>0.25 mmol/l Vitamin C	3
Stage 2 (PGT)	**MCDB 131**:<br>1% Pen/Strep<br>1% L-Glutamine<br>0.5% Fatty acid free BSA<br>1.5 g/l $NaHCO_3$<br>10 mmol/l D-Glucose	50 ng/ml FGF-10<br>0.75 µM Dorsomorphin<br>3 ng/ml WNT-3a<br>0.25 mmol/l Vitamin C	2
Stage 3 (PF)	**DMEM**:<br>1% Pen/Strep<br>4.5 g/l Glucose<br>110 mg/l Sodium pyruvate	50 ng/ml FGF-10<br>200 nM LDN193189<br>0.25 µM SANT-1<br>2 µM Retinoic acid<br>0.25 mmol/l Vitamin C<br>1% B27 w/o Vitamin A	2
Stage 4 (PPs)	**DMEM**:<br>1% Pen/Strep<br>4.5 g/L Glucose<br>110 mg/l Sodium pyruvate	100 ng/ml EGF<br>10 mmol/l Nicotinamide<br>200 nM LDN193189<br>0.25 mmol/l Vitamin C<br>1% B27 w/o Vitamin A	4-6
Stage 5 (EPs)	**MCDB 131:**<br>1% Pen/Strep<br>1% L-Glutamine<br>2% Fatty acid free BSA<br>1.754 g/l NaHCO3<br>3.6 g/l Glucose<br>1:200 ITS-X100<br>10 mg/l Heparin	**Days 1-4**:<br>0.25 µM SANT-1<br>**Days 1-7**:<br>20 ng/ml Betacellulin<br>1 µM G-Secretase inhibitor<br>1 µM T3<br>10 µM ALK5 inhibitor II<br>0.25 mmol/l Vitamin C<br>0.1 µM Retinoic acid	7
Stage 6 (Islet)	**MCDB 131:**<br>1% Pen/Strep<br>1% L-Glutamine<br>2% Fatty acid free BSA<br>1.23 g/l NaHCO3<br>0.45 g/l Glucose<br>1:200 ITS-X100	0.25 mmol/l Vitamin C	7-14

ESM Table 2. List of primers used in the study.

Gene	Forward primer	Reverse primer
APOC3	CTTCATGCAGGGTTACATGAAG	TTTCAGGGAACTGAAGCCATC
ARX	CTGCTGAAACGCAAACAGAGGC	CTCGGTCAAGTCCAGCCTCATG
CDX2	CTGGAGCTGGAGAAGGAGTTTC	ATTTTAACCTGCCTCTCAGAGAGC
CHGA	GAAGAAGGCCCCACTGTAGT	TTCCCAGCTCCATCCACAG
CHGB	CACGCCATTCTGAGAAGAGC	TCTCCTGGCTCTTCAAGGTG
CRYBA2	GATGTGGGTTCCCTCAAAGT	GCTCACCGTAAGTACAGAACTC
ERO1B	TGAACCCAGAGCGTTACACT	GGCGCCAGAGGATTTAAAGG
FEV	GCCTCTCCAAACTCAACCTC	CAAGCTGGGACTGGGGTAG
FFAR1	TGTACCCCAATCTAGGAGGC	GACCCCTTCCCAAGTAACCG
FFAR2	CGGCCTCTGTATGGAGTGAT	CCTGCTCAGTCGTGTTCAAG
FOXA2	GGGAGCGGTGAAGATGGA	TCATGTTGCTCACGGAGGAGTA
GATA6	AAGCGCGTGCCTTCATCA	TCATAGCAAGTGGTCTGGGC
GAPDH	ACGACCACTTTGTCAAGCTCATTTC	GCAGTGAGGGTCTCTCTCTTCCTCT
GCG	CTCTTCACCTGCTCTGTTCTAC	TGGATTTCTCCTCTGTGTCTTG
GCK	GCATCTTCCAGCTCTTCGAC	GGGCTACATTTGAAGGCAGA
IAPP	TTGAGAAGCAATGGGCATCC	GGGTGTAGCTTTCAGATGGTTC
INS	AAGAGGCCATCAAGCAGATCA	CAGGAGGCGCATCCACA
INSM1	TTTGTCTCGTGGTTGGAAGC	CCAAAACAACCCGTACGCTA
IRX1	CAAGAATCCCTACCCCACCA	TCCCCATGTCACCTTGTTCT
IRX2	TCACCAAGATGACCCTCACC	TTCGCTTTTGTTTCTCGGGG
ISL1	CTGTGGACATTACTCCCTCTTAC	GCAACCAACACATAGGGAAATC
KCNJ11	GCGCTTTGTGCCCATTGTA	TTGACGGTGTTGCCAAACTTG
LMX1A	TCCTAGCCTTGGAGAAGCAACT	CAGTGACTGGAGCAGAGAGAA
LMX1B	ACCTCCTTAACCAGCCTCAG	GCATGGAGTAGAGCCGGTC
MAFB	GGAGAATGAGAAGACGCAGC	GTTTCTCGCACTTGACCTTGT
NEUROD1	GCCCCAGGGTTATGAGACTAT	GAGAACTGAGACACTCGTCTGT
NEUROG3	GGCTGTGGGTGCTAAGGGTAAG	CAGGGAGAAGCAGAAGGAACAA
NKX2.2	AAACCATGTCACGCGCTCA	GGCGTTGTACTGCATGTGCT
NKX6.1	GGGCTCGTTTGGCCTATTCGTT	CCACTTGGTCCGGCGGTTCT
ONECUT1	GGACCTCAAGATAGCAGGTTTAT	CAGAATGCAGGTGAGCTAAGT
ONECUT2	GCCATCTTCAAGGAGAACAAAC	CGTTCATGAAGAAGTTGCTGAC
PAX4	AGCAGAGGCACTGGAGAAAGAGTT	CAGCTGCATTTCCCACTTGAGCTT
PAX6	GCGGAAGCTGCAAAGAAATAG	GGGCAAACACATCTGGATAATG
PCSK1	TCACACATGGGGAGAGAACC	TCCCGTGCAAAATCAGCTTC
PCSK2	CGGGTTCCTCTTCTGTGTCA	GACTCCAAAGCCGTGTTCTG
PDX1	CGTCCAGCTGCCTTTCCCAT	CCGTGAGATGTACTTGTTGAATAGGA
PPY	AGGTGCTCGCTTGGTCTAGTG	ACCCAGCAGTGGCTGTAGTAAC
PTF1A	CCAGAAGGTCATCATCTGCC	AGAGAGTGTCCTGCTAGGGG
PTPRN2	ACTGAGGATGTGGAGAAGGC	TGAGTTTGCTTTTCGACCCG

RFX3	AGGAACACAAACTGGACCCA	AGAGGGGAATCTGGCTTGAC
RFX3 genomic DNA	CAACAACAGGTACAGCAGGT	GATTCAGATGGGCGTCACAG
RFX6	GTCGATGCATGGCTTGGACT	TGGGCCATAGCTAGACGGTG
SCG3	GGGACTCCTTTAACCGCTGA	TCCAGTGTATGTGCTTGGCT
SIX3	GCGACTCGGAATGTGATGTAT	GGAGAAGGAAGAGGAGGAAGA
SLC18A1	ATGGTCATCACTGGGGTCAT	GGCTTCTGGGTTGCATACAT
SLC30A8	TGGGAGTCTTGCTGTTGTCA	CATCCAAATGTCAGCCGCTT
SOX9	GACTACACCGACCACCAGAACTCC	GTCTGCGGGATGGAAGGGA
SST	AGCTGCTGTCTGAACCCAAC	CCATAGCCGGGTTTGAGTTA
TPH1	CCCTTCTATACCCCAGAGCC	CCAAGAGAAGCCAAGCCAAT
TXNIP	GATACCCCAGAAGCTCCTCC	TGAACTTGAACTCAGGGGCA
UCN3	GATGGGCTTGGCTTTGTAGA	GGAGGGAAGTCCACTCTCG

ESM Table 3. List of antibodies used in the study.

Antibody	Catalog number	Company	RRID	Dilution
Anti-β-Actin	Sc-47778	Santa Cruz Biotechnology	AB_626632	1:10,000 (WB)
Anti-CDX2	ab76541	Abcam	AB_1523334	1:2000 (WB)
Anti-FEV	25058-1-AP	Proteintech	AB_2879877	1:1000 (IF)
Anti-FOXA2	3143	Cell Signaling Technology	AB_2104878	1:1000 (IF)
Anti-OCT4	4286S	Cell Signaling Technology	AB_1904076	1:500 (IF)
Anti-NANOG	9656s	Cell Signaling Technology	AB_1658242	1:500 (IF)
Anti-SSEA4	9656s	Cell Signaling Technology	AB_1658242	1:500 (IF)
Anti-TRA-1-60	9656s	Cell Signaling Technology	AB_1658242	1:500 (IF)
Anti-SOX17	AF1924	R & D Systems	AB_355060	1:2000 (IF)
Anti-SOX9	HPA001758	Sigma	AB_1080067	1:1000 (IF), 1:4000 (WB)
Anti-GATA6	AF1700	R & D Systems	AB_2108901	1:1000 (IF)
Anti-PDX1	ab47308	Abcam	AB_777178	1:1000 (IF), 1:100 (FACS)
Anti-NKX6.1	F55A12	DSHB	AB_532379	1:2000 (IF), 1:100 (FACS)
Anti-NKX2.2	74.5A5-c	DSHB	AB_531794	1:2000 (IF)
Anti-NEUROG3	AF3444	R & D Systems	AB_2149527	1:1000 (IF)

Anti-RFX3	NBP1-86301	Novus Biologicals	AB_11019457	1:1000 (IF), 1:500 (WB)
Anti-CHGA	MA5-14536	Invitrogen	AB_10978165	1:2000 (IF)
Anti-CHGA	MA5-13096	Invitrogen	AB_10987033	1:2000 (IF)
Anti-TXNIP	ab215366	Abcam	–	1:2000 (WB)
Anti-INS	GN-ID4-s	DSHB	AB_2255626	1:2000 (IF), 1:100 (FACS)
Anti-GCG	G2654	Sigma	AB_259852	1:2000 (IF)
Anti-SST	MAB354	Millipore	AB_2255365	1:2000 (IF)
Anti-UCN3	HPA038281	Sigma	AB_10672408	1:1000 (IF)
Anti-PPY	ab113694	Abcam	AB_11156699	1:1000 (IF)
Anti-GHRL	5992	BioVision	AB_2111582	1:1000 (IF)
Anti-HA Tag	26183	Invitrogen	–	1:500 (IF)
Anti-Ki67 488 conjugated	561165	BD Bioscience	AB_10611866	1:500 (IF)
Anti-CHYMOTRYPSIN	MAB1476	Millipore	AB_2261190	1:500 (IF)
Anti-SLC18A1	HPA063797	Sigma Aldrich	AB_2685125	1:500 (IF)
Alexa Fluor 488 anti-rabbit IgG	A21206	Invitrogen	AB_2535792	1:500 (IF, FACS)
Alexa Fluor 568 anti-rabbit IgG	A10042	Invitrogen	AB_2534017	1:500 (IF)
Alexa Fluor 488 anti-mouse IgG	A21202	Invitrogen	AB_141607	1:500 (IF, FACS)
Alexa Fluor 594 anti-mouse IgG	A32744	Invitrogen	AB_2762826	1:500 (IF)
Alexa Fluor 647 anti-mouse IgG	A31571	Invitrogen	AB_162542	1:500 (FACS)
Alexa Fluor 488 anti-sheep IgG	A11015	Invitrogen	AB_2534082	1:500 (IF)
Alexa Fluor 488 anti-guinea pig IgG	A11073	Invitrogen	AB_2534117	1:500 (IF)
Alexa Fluor 647 anti-guinea pig IgG	A-21450	Invitrogen	AB_2535867	1:500 (FACS)
Alexa Fluor 488 anti-goat IgG	A-11055	Invitrogen	AB_2534102	1:500 (IF)
Alexa Fluor 568 anti-guinea pig IgG	A-11077	Invitrogen	AB_2534121	1:500 (IF, FACS)
Peroxidase AffiniPure Donkey anti-Rabbit IgG (H+L)	711-035-152	Jackson ImmunoResearch Laboratories	AB_10015282	1:10,000 (WB)
Peroxidase AffiniPure Donkey anti-Mouse IgG (H+L)	715-035-150	Jackson ImmunoResearch Laboratories	AB_2340770	1:10,000 (WB)
Peroxidase-AffiniPure Donkey Anti-Guinea Pig IgG (H+L)	706-035-148	Jackson ImmunoResearch Laboratories	AB_2340447	1:10,000 (WB)

ESM Table 4. Top differentially expressed genes (DEGs) (Log_2 FC < -1, Adj *p*-value < 0.05) downregulated in *RFX3* KO1 and *RFX3* KO2 at pancreatic progenitor (PP) stage compared to WT.

**Gene ID**	**Log_2 Fold Change**	**Adj *p*-value**
GCG	-9.432	0.00E+00
UCN3	-8.572	1.39×10^{-14}
SST	-6.520	7.25×10^{-184}
KCNK16	-5.536	$6.51 \times 10^{--58}$
INS	-5.294	1.43×10^{-79}
IL20RA	-5.211	7.59×10^{-49}
ARX	-5.187	4.51×10^{-58}
GHRL	-4.998	8.92×10^{-92}
CNGA3	-4.907	3.81×10^{-25}
CRYBA2	-4.876	2.66×10^{-83}
GPR119	-4.786	1.66×10^{-61}
UCA1	-4.734	2.98×10^{-2}
ABCC8	-4.433	1.14×10^{-33}
VSTM2L	-4.306	1.32×10^{-44}
ISL1	-4.288	9.78×10^{-66}
CRH	-4.210	2.60×10^{-20}
BRINP2	-4.168	5.50×10^{-21}
ERICH3	-4.063	2.50×10^{-18}
GAD2	-3.990	5.51×10^{-28}
CACNA1A	-3.976	1.15×10^{-35}
NSG2	-3.965	3.01×10^{-9}
IRX1	-3.886	1.31×10^{-11}
FGF14	-3.870	3.13×10^{-28}
MAFB	-3.823	1.42×10^{-61}
PSCA	-3.790	3.30×10^{-8}
GJD2	-3.745	2.97×10^{-8}
ATP2A3	-3.739	2.09×10^{-70}
KCNH6	-3.651	3.39×10^{-41}
PERCC1	-3.615	1.68×10^{-18}
ADGRA1	-3.578	1.63×10^{-12}
PAX6	-3.532	4.98×10^{-14}
LHFPL4	-3.521	8.21×10^{-31}
PTPRN	-3.492	4.23×10^{-48}
TRPM3	-3.479	4.08×10^{-19}

SEZ6L	-3.478	1.10×10^{-80}
LY6H	-3.442	1.15×10^{-8}
RGS6	-3.408	2.47×10^{-18}
TNR	-3.355	1.25×10^{-14}
C11orf87	-3.305	1.65×10^{-20}
SSTR2	-3.228	1.60×10^{-51}
PTPRN2	-3.194	1.19×10^{-82}
NECAB2	-3.189	7.33×10^{-16}
SYT5	-3.169	7.54×10^{-9}
NEGR1	-3.104	7.90×10^{-14}
GCK	-3.051	4.59×10^{-15}
ST6GALNAC5	-3.018	2.83×10^{-19}
ASCL2	-2.978	1.46×10^{-8}
SMIM32	-2.974	2.52×10^{-16}
RYR1	-2.964	7.14×10^{-18}
UNC5A	-2.950	2.01×10^{-17}
PCSK1N	-2.892	1.08×10^{-20}
ASCL1	-2.890	7.40×10^{-9}
TMEM196	-2.852	5.14×10^{-18}
THSD4	-2.832	3.76×10^{-51}
SGCD	-2.823	1.67×10^{-21}
KCNK17	-2.808	1.14×10^{-10}
GRIN3A	-2.790	2.29×10^{-25}
GC	-2.782	2.11×10^{-25}
AQP10	-2.780	2.53×10^{-13}
FFAR2	-2.777	1.95×10^{-13}
TMEM130	-2.765	7.54×10^{-7}
CHST8	-2.761	1.78×10^{-14}
CFC1	-2.753	4.00×10^{-6}
GDAP1L1	-2.744	8.47×10^{-14}
PKHD1L1	-2.718	9.71×10^{-11}
KIF19	-2.711	4.51×10^{-15}
UNC13A	-2.710	1.36×10^{-18}
USH2A	-2.700	2.23×10^{-14}
GPBAR1	-2.696	1.03×10^{-13}
KCND3	-2.679	1.79×10^{-39}
FSTL5	-2.655	1.21×10^{-33}

CAMK2B	-2.648	1.41×10^{-22}
MARCHF4	-2.603	6.22×10^{-8}
SCG3	-2.599	1.00×10^{-17}
SYP	-2.599	1.86×10^{-15}
GRIK1	-2.596	2.03×10^{-8}
CHGB	-2.580	2.04×10^{-22}
PDE2A	-2.578	1.66×10^{-15}
RIMBP2	-2.568	2.05×10^{-41}
BAIAP3	-2.565	4.69×10^{-54}
LRRC10B	-2.548	5.69×10^{-9}
CLDN18	-2.539	2.29×10^{-53}
GNAO1	-2.538	5.87×10^{-19}
ERO1B	-2.535	5.07×10^{-42}
CALY	-2.522	1.07×10^{-17}
ST18	-2.516	2.13×10^{-13}
SLC7A14	-2.506	3.72×10^{-14}
VWA5B2	-2.491	1.95×10^{-53}
GNG2	-2.487	5.35×10^{-15}
GDPD2	-2.485	1.43×10^{-9}
CELF3	-2.479	1.35×10^{-45}
CDK5R2	-2.466	2.40×10^{-14}
ASIC4	-2.463	6.14×10^{-9}
NEUROD1	-2.455	2.66×10^{-31}
RASGRF2	-2.455	3.55×10^{-20}
STUM	-2.446	7.78×10^{-10}
KCNB1	-2.434	1.87×10^{-9}
PLCXD3	-2.361	3.97×10^{-11}
PCSK1	-2.345	7.62×10^{-28}
TENM2	-2.328	4.65×10^{-10}
FBXL16	-2.312	1.26×10^{-19}
GRAMD2A	-2.311	2.30×10^{-6}
AMER3	-2.291	8.74×10^{-12}
KCNJ6	-2.287	5.70×10^{-18}
C22orf42	-2.285	1.21×10^{-33}
KCNH3	-2.278	2.23×10^{-22}
RAB26	-2.274	6.37×10^{-71}
RUNDC3A	-2.272	1.94×10^{-20}

SPTBN4	-2.253	2.12×10^{-6}
POU2F2	-2.235	1.01×10^{-9}
UNC80	-2.231	1.01×10^{-24}
CHGA	-2.230	9.70×10^{-17}
DPEP1	-2.229	7.46×10^{-14}
DISP2	-2.207	3.90×10^{-25}
ELAVL4	-2.201	8.11×10^{-8}
IRX2	-2.197	1.41×10^{-12}
ANKS1B	-2.171	2.75×10^{-22}
KCNMA1	-2.169	1.42×10^{-8}
C1QL1	-2.163	2.68×10^{-19}
PGM5	-2.158	1.91×10^{-27}
AMPH	-2.158	1.14×10^{-12}
MGAM2	-2.157	1.57×10^{-13}
TUNAR	-2.150	9.11×10^{-23}
CPLX2	-2.142	3.00×10^{-6}
TSPAN1	-2.118	5.02×10^{-8}
FEV	-2.111	1.68×10^{-16}
NRXN1	-2.093	2.89×10^{-10}
UBE2QL1	-2.092	1.24×10^{-7}
RUNX1T1	-2.084	3.75×10^{-14}
CHRNB2	-2.069	5.27×10^{-10}
AK5	-2.062	1.14×10^{-18}
NFASC	-2.051	8.78×10^{-28}
KCNJ5	-2.048	3.37×10^{-5}
ATCAY	-2.046	2.76×10^{-12}
RAPGEF4	-2.038	1.44×10^{-21}
SVOP	-2.030	1.05×10^{-6}
DLL4	-2.024	3.88×10^{-7}
SYT4	-2.014	2.15×10^{-12}
ADAM12	-1.996	1.17×10^{-7}
CORO2B	-1.985	6.86×10^{-10}
ADCY2	-1.979	8.36×10^{-18}
NEUROG3	-1.972	1.29×10^{-18}
CTNNA2	-1.968	1.68×10^{-16}
NKX2-2	-1.960	9.99×10^{-34}
SPOCK3	-1.957	9.71×10^{-14}

SLIT1	-1.952	3.24×10^{-16}
SLC8A1	-1.952	2.62×10^{-5}
RAP1GAP2	-1.940	5.64×10^{-28}
INSM1	-1.939	1.01×10^{-18}
MMRN1	-1.935	3.07×10^{-3}
ADAMTS2	-1.931	1.60×10^{-7}
SSTR1	-1.922	2.57×10^{-18}
MAP6	-1.920	1.32×10^{-34}
SLC6A4	-1.920	6.91×10^{-12}
SPOCK1	-1.911	1.66×10^{-3}
SLC5A9	-1.900	1.73×10^{-13}
CNIH2	-1.899	2.29×10^{-25}
DEPP1	-1.891	1.45×10^{-10}
PNMA2	-1.891	1.31×10^{-18}
SYT7	-1.882	2.09×10^{-19}
MANEAL	-1.878	1.08×10^{-6}
LMX1B	-1.873	1.76×10^{-56}
CELA3A	-1.868	1.11×10^{-7}
RAB3C	-1.866	5.33×10^{-16}
DUSP26	-1.864	3.40×10^{-10}
DDC	-1.864	2.64×10^{-28}
SEC14L5	-1.863	1.92×10^{-4}
MSC	-1.858	6.93×10^{-11}
NPY5R	-1.857	2.61×10^{-3}
LGALS12	-1.849	1.03×10^{-8}
CACNA1C	-1.840	1.23×10^{-15}
ELAPOR1	-1.829	2.39×10^{-37}
STMN2	-1.821	1.74×10^{-9}
CDHR3	-1.821	1.71×10^{-11}
HPSE	-1.810	1.58×10^{-22}
CKMT1B	-1.803	1.73×10^{-4}
LMO3	-1.800	2.33×10^{-18}
DISP3	-1.775	1.12×10^{-11}
CACNA1B	-1.775	3.69×10^{-15}
MAPK11	-1.768	1.35×10^{-12}
PNPLA7	-1.764	2.85×10^{-10}
LINGO2	-1.756	1.02×10^{-13}

RSAD2	-1.756	3.67 x 10^{-7}
ADAMTS5	-1.753	4.21 x 10^{-7}
HEPACAM2	-1.746	4.49 x 10^{-18}
FAM181B	-1.709	1.66 x 10^{--9}
TPPP3	-1.701	1.64 x 10^{-7}
DRD2	-1.700	4.64 x 10^{-3}
CPA1	-1.698	2.16 x 10^{-11}
GALR1	-1.686	4.69 x 10^{-10}
TRIM50	-1.683	6.92 x 10^{-6}
C7	-1.669	5.96 x 10^{-4}
SLC26A3	-1.653	4.98 x 10^{-3}
GIPR	-1.641	8.62 x 10^{-16}
AP3B2	-1.641	1.69 x 10^{-33}
HS3ST4	-1.635	2.40 x 10^{-10}
UNC79	-1.634	5.08 x 10^{-16}
CD177	-1.627	1.20 x 10^{-9}
IGSF10	-1.623	6.84 x 10^{-13}
CGA	-1.619	1.94 x 10^{-5}
SLC35G2	-1.618	3.30 x 10^{-8}
PCSK2	-1.613	5.29 x 10^{-4}
PPP1R1A	-1.609	2.79 x 10^{-11}
SNAP91	-1.608	1.12 x 10^{-9}
GATM	-1.608	4.01 x 10^{-16}
RGS9	-1.606	6.81 x 10^{-12}
CNNM1	-1.604	6.45 x 10^{-17}
NYAP1	-1.586	3.02 x 10^{-8}
GFRA3	-1.585	6.03 x 10^{-11}
SCN3A	-1.584	3.67 x 10^{-7}
DPP6	-1.581	1.30 x 10^{-11}
DNAH5	-1.570	1.40 x 10^{-4}
SCGN	-1.565	1.89 x 10^{-25}
NPHS1	-1.565	7.90 x 10^{-11}
MAMLD1	-1.541	2.59 x 10^{-24}
ENPP1	-1.541	1.43 x 10^{-6}
LRRTM1	-1.539	2.04 x 10^{-3}
PLXNA2	-1.536	1.18 x 10^{-20}
ACVR1C	-1.535	5.97 x 10^{-9}

PLD5	-1.534	6.54 x 10^{-3}
CTSE	-1.532	3.88 x 10^{-4}
MYOCD	-1.529	2.74 x 10^{-2}
BSN	-1.522	2.76 x 10^{-18}
CNTNAP4	-1.522	1.89 x 10^{-6}
TMOD1	-1.520	5.05 x 10^{-18}
SNED1	-1.514	7.52 x 10^{-9}
CDH12	-1.514	3.19 x 10^{-7}
SV2B	-1.511	2.91 x 10^{-5}
RGS4	-1.509	4.88 x 10^{-6}
SSTR3	-1.499	9.73 x 10^{-4}
FUT2	-1.497	4.51 x 10^{-6}
CRB2	-1.496	6.21 x 10^{-11}
IGFBPL1	-1.495	8.24 x 10^{-6}
SLC25A53	-1.491	6.49 x 10^{-15}
SLC38A3	-1.488	1.94 x 10^{-63}
PDZD2	-1.485	2.61 x 10^{-11}
PRUNE2	-1.480	1.6 x 10^{-16}
ASTN1	-1.475	1.41 x 10^{-2}
BAALC	-1.467	3.04 x 10^{-11}
GRIA2	-1.467	1.08 x 10^{-4}
KIF5A	-1.466	7.30 x 10^{-14}
MLXIPL	-1.461	4.89 x 10^{-12}
KCNK3	-1.460	2.83 x 10^{-6}
PSD	-1.459	4.67 x 10^{-9}
PLPPR1	-1.452	3.32 x 10^{-4}
NEURL1	-1.450	9.42 x 10^{-13}
LGI2	-1.443	4.01 x 10^{-7}
LRP1B	-1.440	5.6 x 10^{-6}
NOVA2	-1.435	7.05 x 10^{-3}
NOL4	-1.434	5.29 x 10^{-7}
GP2	-1.424	2.81 x 10^{-7}
SMPD3	-1.424	3.33 x 10^{-6}
ZNF506	-1.420	2.77 x 10^{-15}
MYPN	-1.419	6.19 x 10^{-9}
XKR7	-1.417	1.57 x 10^{-7}
MAPK8IP2	-1.412	1.63 x 10^{-17}

RASA4B	-1.406	3.44×10^{-14}
ADAMTSL1	-1.406	1.61×10^{-9}
KCNH2	-1.405	2.80×10^{-17}
ADGRF3	-1.388	9.74×10^{-5}
PRKCG	-1.387	1.09×10^{-3}
FMN2	-1.384	1.23×10^{-6}
SCG2	-1.380	7.91×10^{-6}
FRRS1L	-1.376	4.31×10^{-15}
CADM2	-1.372	2.06×10^{-5}
RHOH	-1.370	1.32×10^{-3}
ST8SIA5	-1.368	2.38×10^{-2}
HEYL	-1.368	3.67×10^{-7}
VLDLR	-1.365	4.66×10^{-6}
SLC25A12	-1.353	4.95×10^{-10}
GRHL3	-1.349	2.18×10^{-9}
NAV3	-1.347	6.02×10^{-5}
SMIM5	-1.339	4.30×10^{-3}
TUBA4A	-1.332	6.46×10^{-17}
DIRAS3	-1.328	1.59×10^{-2}
RTL9	-1.327	4.77×10^{-6}
STX1A	-1.327	4.86×10^{-23}
RIMS2	-1.326	1.11×10^{-11}
KIAA0319	-1.323	4.19×10^{-4}
TEKT2	-1.323	2.41×10^{-11}
AMHR2	-1.314	2.49×10^{-15}
BTBD11	-1.313	7.22×10^{-7}
CPA4	-1.312	6.79×10^{-6}
QPCT	-1.311	6.03×10^{-13}
SPAG6	-1.305	7.53×10^{-6}
MNX1	-1.300	2.93×10^{-19}
CLMN	-1.291	5.64×10^{-24}
TACR1	-1.287	7.69×10^{-4}
GAL	-1.286	5.37×10^{-3}
TAGLN3	-1.285	1.44×10^{-6}
ADORA2A	-1.279	2.44×10^{-24}
RASA4	-1.277	4.19×10^{-4}
ACSL1	-1.272	3.08×10^{-27}

IL32	-1.268	4.65 x 10^{-4}
GFRA1	-1.263	6.19 x 10^{-5}
ZFPM2	-1.256	2.88 x 10^{-12}
TTLL6	-1.249	3.78 x 10^{-4}
NANOS1	-1.247	8.41 x 10^{-10}
KIRREL2	-1.244	1.72 x 10^{-3}
GABRB3	-1.243	1.47 x 10^{-16}
VGF	-1.234	4.39 x 10^{-4}
HTRA1	-1.229	5.79 x 10^{-3}
CELF4	-1.229	1.04 x 10^{-10}
RTL1	-1.225	1.04 x 10^{-3}
CASZ1	-1.224	7.24 x 10^{-6}
LSAMP	-1.221	5.62 x 10^{-8}
FNDC11	-1.215	1.69 x 10^{-2}
STC2	-1.212	3.17 x 10^{-12}
SLC16A12	-1.208	5.80 x 10^{-5}
RDH12	-1.206	2.24 x 10^{-6}
POMC	-1.204	8.80 x 10^{-3}
SLC2A14	-1.202	1.87 x 10^{-13}
XKR4	-1.199	3.38 x 10^{-9}
PPP1R3C	-1.187	2.64 x 10^{-8}
SRRM4	-1.177	2.11 x 10^{-7}
RASGRP1	-1.170	4.68 x 10^{-6}
TM4SF4	-1.169	2.09 x 10^{-5}
HYDIN	-1.168	8.68 x 10^{-9}
ZDHHC11B	-1.164	2.01 x 10^{-8}
ATP8A1	-1.160	7.50 x 10^{-7}
TMEM132B	-1.158	4.37 x 10^{-3}
PTF1A	-1.156	2.82 x 10^{-3}
KCNJ11	-1.152	5.83 x 10^{-4}
RASSF6	-1.148	8.22 x 10^{-7}
RFX6	-1.144	1.63 x 10^{-11}
TTBK1	-1.139	1.19 x 10^{-8}
FAM167A	-1.136	4.21 x 10^{-5}
CADPS	-1.135	1.04 x 10^{-8}
CAMK1D	-1.134	2.94 x 10^{-6}
PDE1C	-1.133	1.14 x 10^{-3}

TLE6	-1.127	1.81×10^{-7}
KCNV1	-1.123	2.20×10^{-2}
KCNQ2	-1.122	2.12×10^{-2}
SCN5A	-1.121	7.96×10^{-6}
OSGIN1	-1.121	1.04×10^{-3}
LRRC24	-1.117	9.66×10^{-3}
CPA2	-1.116	1.58×10^{-3}
GNG4	-1.107	1.23×10^{-8}
ACACB	-1.106	2.15×10^{-8}
ANGPT2	-1.102	2.44×10^{-2}
ERN1	-1.101	2.77×10^{-7}
NBEAL2	-1.098	1.02×10^{-32}
EGLN3	-1.094	5.04×10^{-3}
UBD	-1.090	1.57×10^{-2}
PLXNC1	-1.089	7.32×10^{-19}
WNT9A	-1.089	2.96×10^{-4}
CARMIL3	-1.086	6.37×10^{-4}
WDR17	-1.081	1.97×10^{-4}
ECE1	-1.079	9.82×10^{-8}
C2CD2L	-1.079	5.10×10^{-5}
SMARCA2	-1.072	1.08×10^{-10}
STXBP5L	-1.072	3.63×10^{-7}
COPG2IT1	-1.071	1.14×10^{-5}
PDK3	-1.069	$1.53 \times 10^{-}$
STAC	-1.068	1.04×10^{-8}
C4A	-1.062	2.94×10^{-6}
HSFX2	-1.059	1.14×10^{-3}
CD82	-1.057	1.81×10^{-7}
H2BC21	-1.054	2.20×10^{-2}
APC2	-1.053	2.12×10^{-2}
MARCHF8	-1.051	7.96×10^{-6}
ELMO1	-1.046	1.04×10^{-3}
MAPRE3	-1.044	9.66×10^{-3}
ERP27	-1.043	1.58×10^{-3}
RIMKLA	-1.040	1.55×10^{-6}
GCNT1	-1.034	3.89×10^{-8}
SPATA13	-1.034	1.22×10^{-21}

PPFIA3	-1.034	5.47×10^{-6}
ALDH1A1	-1.027	8.30×10^{-6}
CKMT1A	-1.027	1.68×10^{-2}
CACNA1I	-1.025	4.26×10^{-3}
CKMT2	-1.024	1.03×10^{-5}
SNAP25	-1.023	3.24×10^{-8}
MAPK15	-1.023	8.65×10^{-3}
B3GALT4	-1.020	4.50×10^{-3}
GRIK2	-1.007	9.17×10^{-4}
SOX1	-1.006	5.29×10^{-4}
SPOCK2	-1.000	1.17×10^{-2}

ESM Table 5. Top differentially expressed genes (DEGs) (Log_2 FC > 1, Adj *p*-value < 0.05) upregulated in *RFX3* KO1 and *RFX3* KO2 at pancreatic progenitor (PP) stage compared to WT.

Gene ID	Log_2 Fold Change	Adj *p*-value
APOA4	2.695	1.76×10^{-20}
ITIH3	2.585	6.96×10^{-5}
SLC22A7	2.459	2.07×10^{-3}
HRG	2.447	3.62×10^{-9}
ALDOB	2.359	3.19×10^{-11}
FGF18	2.252	3.44×10^{-2}
KNG1	2.131	9.10×10^{-9}
MAT1A	2.128	1.62×10^{-17}
ALB	2.030	5.39×10^{-25}
IP6K3	2.025	4.73×10^{-2}
RBP2	1.945	2.94×10^{-6}
SI	1.897	3.23×10^{-23}
ITIH1	1.892	2.46×10^{-4}
SPP1	1.889	4.86×10^{-4}
SERPINA7	1.823	1.08×10^{-14}
LRRK2	1.737	5.35×10^{-15}
HMGCS2	1.710	1.44×10^{-5}
TPH1	1.686	4.56×10^{-3}
PCAT1	1.637	3.94×10^{-3}
FGG	1.621	7.75×10^{-37}

UGT2B10	1.615	3.33×10^{-6}
FAM151A	1.610	9.32×10^{-13}
APOC2	1.602	7.90×10^{-6}
URAD	1.585	3.70×10^{-3}
FGA	1.568	1.34×10^{-27}
FETUB	1.564	5.04×10^{-3}
ANGPTL3	1.519	1.73×10^{-3}
GUCY2C	1.509	5.36×10^{-8}
TDO2	1.496	8.06×10^{-16}
APOC3	1.496	1.27×10^{-5}
METTL7A	1.492	3.13×10^{-4}
ITIH2	1.483	2.82×10^{-4}
MMP1	1.481	2.58×10^{-6}
CALB1	1.458	6.53×10^{-6}
TF	1.452	6.60×10^{-9}
CREB3L3	1.442	1.63×10^{-9}
SMLR1	1.436	1.39×10^{-15}
HRH2	1.436	1.22×10^{-8}
ANGPT1	1.434	7.18×10^{-11}
RFTN2	1.420	2.75×10^{-5}
ACSL5	1.400	1.07×10^{-12}
AKR1D1	1.385	9.63×10^{-26}
KCNJ13	1.379	5.82×10^{-6}
GSTA2	1.377	8.27×10^{-43}
PIK3C2G	1.369	2.16×10^{-7}
HPX	1.364	1.54×10^{-10}
C20orf204	1.357	1.02×10^{-4}
COL25A1	1.354	8.74×10^{-3}
SLITRK3	1.348	3.76×10^{-6}
AHSG	1.334	2.68×10^{-6}
HSD17B2	1.324	1.03×10^{-9}
ECM2	1.310	1.63×10^{-5}
PLG	1.307	7.92×10^{-5}
CLDN2	1.307	1.88×10^{-4}
GSTA1	1.287	8.11×10^{-14}
MEP1A	1.284	1.76×10^{-16}
MGAM	1.281	3.57×10^{-4}

KLB	1.280	2.39×10^{-4}
GPM6A	1.278	4.67×10^{-3}
SERPINA6	1.277	2.70×10^{-3}
APOB	1.271	9.40×10^{-7}
GBA3	1.260	7.91×10^{-5}
ARHGAP23	1.257	5.22×10^{-4}
PTCHD4	1.256	2.86×10^{-2}
DPYS	1.255	3.21×10^{-11}
L1TD1	1.248	1.53×10^{-16}
FGB	1.247	2.11×10^{-26}
CUBN	1.247	3.40×10^{-17}
SCHIP1	1.241	1.75×10^{-2}
COL11A1	1.240	2.70×10^{-5}
FMO1	1.240	1.84×10^{-5}
CFTR	1.217	5.00×10^{-9}
AFP	1.198	1.03×10^{-16}
EGFLAM	1.188	2.62×10^{-9}
FABP1	1.186	2.41×10^{-4}
GDNF	1.182	4.50×10^{-3}
NRGN	1.179	1.18×10^{-9}
PRAP1	1.168	7.12×10^{-3}
SP8	1.168	1.72×10^{-4}
PALMD	1.166	1.17×10^{-2}
ALDH1L1	1.166	5.13×10^{-5}
UGT2B7	1.153	1.69×10^{-5}
CA2	1.144	3.41×10^{-21}
XYLB	1.140	1.80×10^{-4}
HOGA1	1.134	2.76×10^{-4}
SERPINC1	1.129	3.62×10^{-2}
MAOB	1.125	6.85×10^{-5}
A2M	1.120	6.08×10^{-4}
LEAP2	1.118	1.60×10^{-2}
EGF	1.117	3.83×10^{-2}
FMO5	1.106	3.69×10^{-3}
IYD	1.100	1.91×10^{-2}
LIPC	1.097	3.16×10^{-6}
PCDHA10	1.094	3.78×10^{-5}

METTL7B	1.092	1.03×10^{-4}
RBP4	1.088	1.67×10^{-9}
ITIH4	1.072	5.71×10^{-4}
SOAT2	1.070	6.37×10^{-7}
PLP1	1.066	4.64×10^{-2}
SLC13A5	1.058	3.34×10^{-3}
NDRG1	1.057	3.59×10^{-5}
WNT11	1.054	2.25×10^{-4}
ADAMTS3	1.046	4.60×10^{-2}
INPP5D	1.039	1.58×10^{-2}
SLITRK6	1.032	2.62×10^{-2}
TNFRSF10C	1.026	6.33×10^{-3}
VEPH1	1.019	1.28×10^{-3}
RELN	1.018	3.07×10^{-3}
KYNU	1.014	8.04×10^{-8}
AGT	1.008	9.43×10^{-7}
KLK6	1.006	1.20×10^{-3}
ADAMTS12	1.003	4.13×10^{-8}
DGAT2	1.002	3.33×10^{-4}

ESM Table 6. Top differentially expressed genes (DEGs) (Log_2 FC < -1, Adj *p*-value < 0.05) downregulated in *RFX3* KO1 and *RFX3* KO2 at islet stage (S6) compared to WT.

**Gene ID**	**Log_2 Fold Change**	**Adj *p*-value**
GCG	-6.593	2.47×10^{-102}
IBSP	-5.126	8.43×10^{-13}
SLC30A8	-4.957	5.04×10^{-33}
LRRC53	-4.763	6.70×10^{-26}
IAPP	-4.763	1.73×10^{-19}
INS	-4.611	2.19×10^{-10}
FFAR1	-4.565	1.05×10^{-5}
SST	-4.323	4.55×10^{-45}
PRG4	-3.775	5.34×10^{-29}
SERINC4	-3.554	2.25×10^{-2}
S100B	-3.448	3.42×10^{-6}
BHMT	-3.257	5.23×10^{-4}

KCNK16	-3.183	1.17×10^{-22}
PAX5	-3.123	1.66×10^{-2}
SLC6A17	-3.121	6.08×10^{-14}
PAX6	-2.868	1.85×10^{-12}
FGF17	-2.861	8.93×10^{-8}
FEZF1	-2.856	8.91×10^{-4}
GCK	-2.798	7.32×10^{-10}
ANGPTL1	-2.779	9.07×10^{-3}
CNGA3	-2.751	4.71×10^{-27}
CDH8	-2.713	2.30×10^{-9}
FGF1	-2.710	1.33×10^{-6}
GAP43	-2.673	1.33×10^{-6}
RMST	-2.670	2.17×10^{-19}
MCHR1	-2.656	6.12×10^{-12}
FAP	-2.610	2.27×10^{-11}
FEZF2	-2.594	1.35×10^{-2}
ABCC8	-2.586	1.79×10^{-62}
RGS1	-2.580	1.05×10^{-8}
CACNA1E	-2.557	8.00×10^{-5}
FABP7	-2.550	1.42×10^{-7}
SIX6	-2.544	2.97×10^{-6}
NPTX2	-2.530	2.42×10^{-6}
WNT8B	-2.511	5.75×10^{-3}
LHX2	-2.511	5.17×10^{-3}
SPARCL1	-2.493	3.61×10^{-7}
NTNG2	-2.480	6.29×10^{-6}
EGR3	-2.453	8.64×10^{-4}
PLP1	-2.424	8.02×10^{-4}
KIRREL3	-2.409	6.05×10^{-6}
MEGF10	-2.403	6.98×10^{-4}
TUBB1	-2.397	9.68×10^{-13}
ISL1	-2.385	1.22×10^{-10}
ERO1B	-2.364	2.64×10^{-33}
SYT12	-2.334	6.01×10^{-5}
CFI	-2.325	2.39×10^{-16}
ZMYND10	-2.311	4.96×10^{-6}
TMEM158	-2.307	5.25×10^{-7}
FAM181A	-2.286	3.08×10^{-2}

GABRQ	-2.279	6.24×10^{-5}
WDR49	-2.277	2.12×10^{-2}
SCN1A	-2.268	1.06×10^{-4}
DLX1	-2.264	1.79×10^{-5}
CRB1	-2.260	1.68×10^{-3}
TPPP3	-2.248	4.44×10^{-3}
DOCK10	-2.240	2.00×10^{-9}
BTBD17	-2.229	4.23×10^{-4}
ZIC2	-2.225	2.47×10^{-4}
LMO1	-2.225	9.93×10^{-4}
SUCNR1	-2.218	2.58×10^{-6}
RTL1	-2.211	1.77×10^{-5}
NTN1	-2.207	1.74×10^{-3}
AMER2	-2.207	7.16×10^{-3}
GAD2	-2.203	5.61×10^{-54}
ARX	-2.201	7.06×10^{-4}
LPL	-2.182	1.25×10^{-6}
F13A1	-2.181	1.47×10^{-2}
CORIN	-2.179	1.42×10^{-4}
C6orf118	-2.175	2.81×10^{-2}
ZIC3	-2.161	6.11×10^{-3}
SHC3	-2.153	1.85×10^{-16}
VCAM1	-2.150	1.31×10^{-4}
CYP26C1	-2.142	1.55×10^{-3}
RYR1	-2.140	1.12×10^{-6}
CALB2	-2.131	4.20×10^{-9}
GRIK4	-2.130	8.59×10^{-5}
NEUROG3	-2.128	3.96×10^{-5}
ERICH3	-2.109	1.56×10^{-11}
PTPRO	-2.107	2.49×10^{-5}
PTPRZ1	-2.105	2.04×10^{-5}
GPM6B	-2.087	2.40×10^{-6}
CRIP2	-2.079	1.76×10^{-12}
ZIC1	-2.073	7.92×10^{-4}
EGR2	-2.065	5.76×10^{-10}
SALL3	-2.053	1.46×10^{-5}
NKX6-2	-2.050	1.39×10^{-9}
PDE1A	-2.047	2.78×10^{-4}

FOXG1	-2.043	8.73 x 10^{-3}
EPHA5	-2.032	1.33 x 10^{-4}
USH2A	-2.031	3.78 x 10^{-6}
LY6H	-2.012	4.99 x 10^{-2}
ZIC5	-2.008	1.67 x 10^{-2}
ADD2	-1.997	6.03 x 10^{-3}
APCDD1	-1.994	2.15 x 10^{-9}
MAPK15	-1.985	2.19 x 10^{-28}
SLC17A8	-1.973	1.09 x 10^{-13}
SLC1A2	-1.968	3.92 x 10^{-2}
NSG2	-1.967	4.17 x 10^{-2}
DPP6	-1.962	4.57 x 10^{-8}
NTRK2	-1.951	4.06 x 10^{-10}
SPAG6	-1.951	8.49 x 10^{-10}
KCNK17	-1.950	1.31 x 10^{-6}
MLC1	-1.941	1.99 x 10^{-3}
HYDIN	-1.927	5.41 x 10^{-6}
GRPR	-1.925	1.06 x 10^{-3}
CBLN1	-1.920	3.38 x 10^{-21}
CCL2	-1.919	1.11 x 10^{-3}
KCNA6	-1.915	4.46 x 10^{-8}
C5orf49	-1.912	1.55 x 10^{-7}
IRX1	-1.898	2.43 x 10^{-3}
EFCC1	-1.898	7.84 x 10^{-3}
TMEM179	-1.884	3.50 x 10^{-5}
GALNT17	-1.883	1.88 x 10^{-3}
GAD1	-1.881	1.91 x 10^{-2}
THY1	-1.876	1.88 x 10^{-5}
PCDH15	-1.875	1.29 x 10^{-4}
PREX1	-1.869	2.35 x 10^{-3}
ITGA11	-1.861	4.86 x 10^{-8}
VWC2	-1.860	6.56 x 10^{-5}
AMTN	-1.856	3.09 x 10^{-5}
TEKT2	-1.852	4.46 x 10^{-8}
CABP7	-1.837	2.32 x 10^{-4}
LRRTM2	-1.837	9.33 x 10^{-3}
CNIH2	-1.828	1.20 x 10^{-13}
SLC8A3	-1.828	2.53 x 10^{-6}

APC2	-1.827	2.61×10^{-5}
IRX2	-1.826	3.25×10^{-3}
IFI44L	-1.824	1.77×10^{-2}
RELN	-1.818	1.56×10^{-10}
ZIC4	-1.813	1.16×10^{-2}
LRP8	-1.810	1.00×10^{-8}
FOSB	-1.800	3.15×10^{-4}
GRM4	-1.796	5.40×10^{-7}
TMEM178B	-1.795	5.17×10^{-11}
KAAG1	-1.793	1.98×10^{-2}
CFAP157	-1.789	4.42×10^{-3}
PDZD4	-1.786	4.20×10^{-9}
GRIA1	-1.776	5.34×10^{-6}
SEPTIN3	-1.775	3.41×10^{-4}
STK32B	-1.773	2.43×10^{-4}
B4GALNT1	-1.767	3.11×10^{-4}
LEFTY1	-1.764	8.42×10^{-8}
FGF8	-1.762	1.01×10^{-2}
ENO4	-1.762	5.23×10^{-4}
VAX1	-1.756	4.58×10^{-2}
CD44	-1.754	3.01×10^{-13}
RERG	-1.746	1.53×10^{-8}
SLC32A1	-1.741	9.34×10^{-3}
DCLK1	-1.734	3.88×10^{-6}
MEP1B	-1.733	1.46×10^{-5}
STMN4	-1.729	3.49×10^{-3}
GPM6A	-1.722	3.10×10^{-2}
EVA1C	-1.721	1.40×10^{-2}
SOX1	-1.720	3.05×10^{-3}
SLC6A15	-1.719	1.66×10^{-11}
PNMA8C	-1.717	5.04×10^{-3}
CRB2	-1.710	6.31×10^{-3}
CSPG5	-1.709	7.06×10^{-4}
OTX2	-1.706	2.61×10^{-2}
PCSK1N	-1.705	1.99×10^{-4}
CXCR4	-1.702	3.05×10^{-9}
FGFBP3	-1.698	5.64×10^{-4}
LGALS1	-1.696	1.73×10^{-6}

PTGER3	-1.695	3.33 x 10^{-7}
GFRA3	-1.689	2.89 x 10^{-12}
ARC	-1.684	1.92 x 10^{-2}
TMPRSS5	-1.680	7.40 x 10^{-4}
FIBIN	-1.676	3.49 x 10^{-3}
KCNN3	-1.676	1.34 x 10^{-3}
CHST8	-1.674	4.96 x 10^{-5}
ILDR2	-1.674	5.84 x 10^{-3}
CCDC74B	-1.673	9.66 x 10^{-15}
HAPLN1	-1.672	3.01 x 10^{-10}
POSTN	-1.667	1.08 x 10^{-4}
NFIX	-1.667	6.33 x 10^{-5}
PCDHGB1	-1.663	1.32 x 10^{-2}
MAP3K7CL	-1.658	2.33 x 10^{-5}
SPEF1	-1.658	7.21 x 10^{-4}
ANKRD63	-1.649	1.39 x 10^{-3}
SULF1	-1.644	8.14 x 10^{-4}
FEZ1	-1.641	2.33 x 10^{-5}
PDE4B	-1.630	9.95 x 10^{-4}
ECEL1	-1.624	6.25 x 10^{-11}
SOGA3	-1.624	5.95 x 10^{-4}
PTX3	-1.622	5.89 x 10^{-4}
ADGRL4	-1.619	2.20 x 10^{-4}
RHOJ	-1.605	5.66 x 10^{-5}
GABRG3	-1.604	1.05 x 10^{-3}
KL	-1.598	3.19 x 10^{-4}
PTCH2	-1.598	1.86 x 10^{-2}
CLEC18B	-1.590	1.44 x 10^{-5}
NPHS1	-1.589	3.66 x 10^{-7}
TMEM132B	-1.587	1.21 x 10^{-8}
ADAMTS3	-1.585	2.83 x 10^{-3}
NFIA	-1.583	2.95 x 10^{-5}
CALN1	-1.581	2.19 x 10^{-3}
HGFAC	-1.566	2.93 x 10^{-5}
SCG5	-1.564	3.50 x 10^{-5}
DLX5	-1.561	1.29 x 10^{-2}
LRRIQ1	-1.559	1.75 x 10^{-2}
NOVA2	-1.558	1.90 x 10^{-3}

DMBX1	-1.556	1.83×10^{-4}
PLPPR4	-1.553	1.03×10^{-2}
PLA2G3	-1.551	7.42×10^{-3}
VGF	-1.547	1.14×10^{-3}
SYT5	-1.544	1.33×10^{-6}
RSPO3	-1.541	3.84×10^{-2}
LMO2	-1.534	8.34×10^{-3}
VEGFC	-1.533	5.14×10^{-3}
DCLK2	-1.532	5.08×10^{-5}
CCDC184	-1.532	3.83×10^{-3}
GNG2	-1.531	2.83×10^{-6}
CSMD2	-1.530	3.42×10^{-4}
CCDC81	-1.529	6.01×10^{-5}
PPM1E	-1.524	8.68×10^{-4}
PLXNB3	-1.520	1.12×10^{-2}
JAM2	-1.519	6.31×10^{-4}
PTPRN	-1.518	8.51×10^{-14}
NPAS3	-1.513	2.62×10^{-3}
TRPC4	-1.508	2.16×10^{-3}
ARMC3	-1.508	2.77×10^{-4}
ATP8A2	-1.507	1.39×10^{-3}
COL6A3	-1.506	2.56×10^{-4}
CSPG4	-1.504	9.14×10^{-6}
DCDC1	-1.504	1.47×10^{-4}
PLCXD3	-1.504	4.20×10^{-9}
TNC	-1.497	1.94×10^{-4}
DNAAF3	-1.493	2.10×10^{-3}
MIAT	-1.485	3.40×10^{-8}
ADAMTS14	-1.484	1.02×10^{-4}
ADGRF5	-1.483	7.36×10^{-10}
PRODH2	-1.482	2.89×10^{-3}
SIX3	-1.480	1.21×10^{-2}
PGM5	-1.480	1.23×10^{-5}
OLFM1	-1.478	1.86×10^{-2}
DNAH6	-1.478	3.35×10^{-3}
DKK2	-1.476	2.61×10^{-3}
GRIN2B	-1.472	6.70×10^{-4}
CADM3	-1.467	8.96×10^{-4}

DNAAF1	-1.467	1.12 x 10^{-2}
FGL1	-1.463	1.48 x 10^{-2}
LRRC4	-1.457	4.76 x 10^{-5}
MLIP	-1.454	1.25 x 10^{-2}
GREM1	-1.452	4.15 x 10^{-10}
DPYSL5	-1.452	5.08 x 10^{-3}
CCDC74A	-1.452	4.86 x 10^{-4}
TTYH1	-1.451	5.66 x 10^{-5}
AK5	-1.449	2.08 x 10^{-3}
CELF5	-1.446	2.14 x 10^{-2}
ALKAL2	-1.445	2.95 x 10^{-2}
THBS2	-1.444	3.16 x 10^{-3}
NOG	-1.443	1.13 x 10^{-2}
NLGN4X	-1.437	2.52 x 10^{-6}
DHRS2	-1.436	1.92 x 10^{-15}
DLK1	-1.434	2.48 x 10^{-15}
HMGCLL1	-1.431	2.43 x 10^{-6}
KCNMB1	-1.431	1.84 x 10^{-2}
METRN	-1.425	4.58 x 10^{-13}
ATP1A2	-1.422	1.63 x 10^{-2}
IGSF9B	-1.417	3.51 x 10^{-5}
DLX2	-1.415	4.97 x 10^{-2}
RN7SL2	-1.414	3.95 x 10^{-3}
NCALD	-1.410	1.31 x 10^{-3}
CYP26A1	-1.402	3.89 x 10^{-3}
NCAM1	-1.402	2.33 x 10^{-5}
CLEC11A	-1.402	3.37 x 10^{-3}
ZEB1	-1.401	3.03 x 10^{-2}
TMEM130	-1.394	2.99 x 10^{-2}
CNTNAP3	-1.392	1.26 x 10^{-3}
CHST7	-1.390	8.71 x 10^{-4}
DYRK3	-1.389	1.72 x 10^{-5}
RNF182	-1.385	8.97 x 10^{-4}
CCK	-1.384	1.96 x 10^{-3}
RGMA	-1.380	1.53 x 10^{-2}
ZNF521	-1.378	5.83 x 10^{-3}
TMSB15A	-1.376	1.44 x 10^{-2}
ADAMTSL1	-1.375	9.63 x 10^{-3}

GFAP	-1.373	4.71 x 10^{-3}
DNER	-1.373	1.39 x 10^{-3}
NIM1K	-1.371	9.47 x 10^{-3}
TACR1	-1.371	2.22 x 10^{-4}
BEX1	-1.367	1.92 x 10^{-15}
TMEM145	-1.364	4.00 x 10^{-4}
CDH10	-1.361	9.04 x 10^{-10}
CDO1	-1.360	9.07 x 10^{-4}
ATOH8	-1.356	8.08 x 10^{-3}
COL9A1	-1.352	2.27 x 10^{-2}
LRRC10B	-1.349	1.12 x 10^{-3}
SCN7A	-1.347	9.60 x 10^{-3}
ST6GAL2	-1.343	5.72 x 10^{-3}
PLPPR5	-1.343	4.55 x 10^{-4}
SEMA5B	-1.342	7.92 x 10^{-3}
KCNQ2	-1.332	3.95 x 10^{-2}
BBOX1	-1.332	8.20 x 10^{-3}
SPOCK3	-1.329	5.58 x 10^{-4}
MAPK10	-1.329	4.88 x 10^{-4}
BCL2	-1.324	2.54 x 10^{-2}
ACTG2	-1.324	1.02 x 10^{-3}
PRR7	-1.324	1.44 x 10^{-5}
SHC4	-1.320	1.19 x 10^{-4}
FBXL16	-1.315	1.59 x 10^{-4}
MAP2	-1.309	4.49 x 10^{-11}
CCN3	-1.307	6.59 x 10^{-6}
CTXN1	-1.306	2.39 x 10^{-8}
PRRX1	-1.305	4.40 x 10^{-2}
CALY	-1.304	4.72 x 10^{-4}
ADGRV1	-1.303	1.62 x 10^{-6}
RAPGEF4	-1.300	9.07 x 10^{-8}
ZNF506	-1.299	1.93 x 10^{-6}
ADAM12	-1.296	2.29 x 10^{-2}
EPHB1	-1.296	3.19 x 10^{-3}
HOPX	-1.294	4.72 x 10^{-3}
RPRM	-1.293	3.42 x 10^{-3}
NNAT	-1.290	4.17 x 10^{-7}
CACNG8	-1.288	1.99 x 10^{-3}

DIRAS2	-1.286	1.97×10^{-2}
LOXL2	-1.286	6.95×10^{-9}
KISS1R	-1.282	1.59×10^{-4}
FRZB	-1.280	4.81×10^{-2}
HS6ST3	-1.278	1.71×10^{-10}
LRFN2	-1.277	2.62×10^{-2}
PACRG	-1.273	2.46×10^{-5}
SOX2	-1.271	2.38×10^{-2}
SYT11	-1.262	1.60×10^{-5}
LRRC4B	-1.261	4.65×10^{-2}
UNC5A	-1.260	3.65×10^{-2}
DENND2A	-1.258	2.55×10^{-3}
FGFR1	-1.258	2.17×10^{-10}
GPC2	-1.257	7.28×10^{-9}
PITPNC1	-1.253	3.43×10^{-10}
NUF2	-1.251	2.07×10^{-3}
COL8A1	-1.248	2.59×10^{-2}
ASTN1	-1.247	2.86×10^{-2}
PNMA2	-1.240	4.23×10^{-23}
DNAH7	-1.238	1.33×10^{-2}
STARD9	-1.238	1.91×10^{-3}
RTN1	-1.235	1.90×10^{-3}
MAP6	-1.235	1.79×10^{-5}
RGS16	-1.226	5.49×10^{-6}
PNMT	-1.223	1.56×10^{-3}
ADCYAP1R1	-1.222	1.24×10^{-2}
BGN	-1.222	1.97×10^{-2}
RHPN1	-1.218	5.51×10^{-5}
FAM72B	-1.217	3.06×10^{-2}
VIM	-1.216	2.33×10^{-5}
PIF1	-1.213	1.42×10^{-3}
PAK3	-1.212	9.74×10^{-6}
UBXN10	-1.210	1.93×10^{-5}
BAIAP3	-1.210	1.80×10^{-11}
CCDC160	-1.207	1.15×10^{-2}
KCNJ11	-1.205	2.98×10^{-12}
ELAVL4	-1.198	1.03×10^{-4}
CFTR	-1.197	5.25×10^{-3}

PCDHGC4	-1.197	4.51×10^{-2}
FOS	-1.197	7.44×10^{-4}
CCDC181	-1.194	3.04×10^{-3}
ELAVL3	-1.191	2.90×10^{-2}
NLGN3	-1.191	3.26×10^{-3}
SYNPR	-1.191	3.02×10^{-2}
AMHR2	-1.190	6.88×10^{-3}
GDAP1L1	-1.189	3.07×10^{-3}
SUGCT	-1.186	9.34×10^{-3}
MATN2	-1.184	2.33×10^{-5}
SLC35F1	-1.184	2.77×10^{-2}
COL9A3	-1.183	3.89×10^{-5}
SFTA1P	-1.181	4.44×10^{-2}
MDH1B	-1.180	2.21×10^{-3}
FAM181B	-1.178	3.00×10^{-3}
FXYD2	-1.175	4.46×10^{-7}
GHRL	-1.174	7.29×10^{-3}
CHRNA5	-1.173	2.60×10^{-5}
S1PR3	-1.172	4.61×10^{-4}
HIC1	-1.172	2.63×10^{-3}
ANGPTL4	-1.171	2.77×10^{-4}
PAPPA	-1.171	1.23×10^{-3}
KIF5A	-1.171	4.45×10^{-4}
SUSD4	-1.170	1.12×10^{-3}
KRT4	-1.170	1.79×10^{-2}
SYT2	-1.170	5.18×10^{-4}
LMO3	-1.168	1.13×10^{-2}
CRMP1	-1.168	9.55×10^{-5}
JAM3	-1.166	5.84×10^{-3}
SCG3	-1.164	9.70×10^{-20}
OIP5	-1.164	1.77×10^{-2}
HSF4	-1.162	5.66×10^{-5}
TAGLN3	-1.158	3.43×10^{-2}
LDLRAD4	-1.156	8.36×10^{-7}
CORO1A	-1.154	1.40×10^{-4}
FAM131C	-1.154	5.79×10^{-5}
FHL1	-1.153	5.30×10^{-3}
GLIPR1	-1.150	1.22×10^{-3}

FBLL1	-1.146	6.90 x 10^{-4}
IGSF11	-1.141	6.89 x 10^{-4}
CHN1	-1.140	1.66 x 10^{-3}
CENPW	-1.140	1.99 x 10^{-5}
MAPK11	-1.139	3.29 x 10^{-3}
SLC6A2	-1.138	2.86 x 10^{-2}
NR2F1	-1.136	2.14 x 10^{-2}
NALCN	-1.135	8.83 x 10^{-3}
TSPOAP1	-1.134	5.26 x 10^{-7}
NRXN1	-1.134	8.03 x 10^{-7}
LRRC24	-1.132	1.07 x 10^{-3}
NAP1L3	-1.132	8.67 x 10^{-5}
DRC7	-1.131	3.16 x 10^{-3}
WNT10A	-1.131	3.83 x 10^{-3}
STMN2	-1.128	2.70 x 10^{-16}
PEG13	-1.124	5.32 x 10^{-3}
MEX3B	-1.124	5.09 x 10^{-3}
EGR1	-1.123	1.27 x 10^{-2}
TRIM9	-1.123	2.09 x 10^{-2}
UNC13A	-1.122	3.41 x 10^{-14}
KCNMA1	-1.121	2.57 x 10^{-7}
CYP1B1	-1.118	7.47 x 10^{-4}
PPIL6	-1.117	1.71 x 10^{-3}
RRAD	-1.117	6.97 x 10^{-3}
DPYSL4	-1.117	5.34 x 10^{-6}
BMERB1	-1.115	1.25 x 10^{-5}
RAB36	-1.114	2.60 x 10^{-6}
ADGRB3	-1.109	5.69 x 10^{-4}
ST8SIA2	-1.105	9.38 x 10^{-3}
CCNA1	-1.105	1.04 x 10^{-3}
GFPT2	-1.104	2.44 x 10^{-2}
GRIN2A	-1.101	3.38 x 10^{-4}
EFNB3	-1.101	2.25 x 10^{-4}
ETV1	-1.097	2.43 x 10^{-3}
GRM8	-1.096	8.58 x 10^{-3}
TMEM59L	-1.094	2.05 x 10^{-3}
C8A	-1.093	1.68 x 10^{-2}
ARHGAP33	-1.091	3.48 x 10^{-3}

LHFPL6	-1.088	6.58×10^{-5}
TMEFF1	-1.088	2.80×10^{-2}
ELAVL2	-1.088	2.61×10^{-2}
HCN1	-1.088	2.90×10^{-4}
DPF1	-1.087	1.68×10^{-2}
ATCAY	-1.083	2.14×10^{-2}
EDN3	-1.083	8.64×10^{-3}
PNMA8B	-1.081	1.60×10^{-4}
KCNIP1	-1.081	2.52×10^{-2}
ACTA2	-1.081	8.72×10^{-3}
MAPK8IP1	-1.079	6.41×10^{-11}
SYDE1	-1.079	4.61×10^{-5}
NTM	-1.077	9.47×10^{-4}
TMEM262	-1.076	2.45×10^{-3}
CYTL1	-1.071	1.37×10^{-2}
HAGHL	-1.071	1.58×10^{-3}
DIRAS3	-1.071	8.23×10^{-4}
PLCH2	-1.070	3.91×10^{-3}
LHFPL4	-1.068	1.07×10^{-3}
ZCCHC18	-1.065	3.33×10^{-5}
CADPS	-1.061	2.06×10^{-4}
FAT3	-1.058	8.67×10^{-3}
NANOS1	-1.058	1.38×10^{-3}
CCDC78	-1.054	1.94×10^{-2}
HS3ST3B1	-1.054	3.83×10^{-4}
INA	-1.053	1.15×10^{-9}
RSPH4A	-1.051	5.27×10^{-4}
TMEM151B	-1.051	3.43×10^{-2}
LGR5	-1.051	6.03×10^{-3}
GPR68	-1.048	7.79×10^{-3}
SIX2	-1.045	3.05×10^{-4}
TSNAXIP1	-1.044	4.00×10^{-3}
CENPF	-1.044	5.40×10^{-4}
ANO4	-1.043	8.40×10^{-9}
SYT4	-1.042	2.33×10^{-5}
PCDHB15	-1.038	4.64×10^{-2}
EFCAB12	-1.038	3.45×10^{-2}
TMEM121	-1.037	4.11×10^{-2}

TENM2	-1.037	3.19×10^{-4}
CRYAB	-1.035	2.82×10^{-2}
TRIM46	-1.034	1.08×10^{-3}
PSRC1	-1.030	9.58×10^{-4}
ZNF350	-1.026	5.41×10^{-3}
XKR4	-1.025	1.54×10^{-3}
EDAR	-1.025	1.69×10^{-2}
MAPK4	-1.021	7.05×10^{-8}
MRC2	-1.021	4.30×10^{-6}
SOD3	-1.020	9.45×10^{-4}
PCYT1B	-1.020	1.33×10^{-4}
REC8	-1.020	4.64×10^{-17}
FAM167A	-1.019	3.22×10^{-7}
WDR25	-1.019	7.10×10^{-10}
TNR	-1.017	2.15×10^{-7}
UNC5D	-1.016	6.37×10^{-4}
INSYN1	-1.012	3.73×10^{-2}
RNF150	-1.012	2.08×10^{-3}
FJX1	-1.011	8.01×10^{-9}
GALNT15	-1.009	1.03×10^{-2}
LRRC49	-1.008	7.43×10^{-4}
WDR97	-1.007	8.32×10^{-4}
STMN3	-1.007	1.67×10^{-3}
NOTCH1	-1.006	5.51×10^{-5}
PCDHB5	-1.005	1.43×10^{-2}
TPM2	-1.003	3.86×10^{-5}

ESM Table 7. Top differentially expressed genes (DEGs) (Log_2 FC > 1, Adj *p*-value < 0.05) upregulated in *RFX3* KO1 and *RFX3* KO2 at islet stage (S6) compared to WT.

**Gene ID**	**Log_2 Fold Change**	**Adj *p*-value**
MT1H	3.333	5.18×10^{-4}
ALOX15B	3.250	1.50×10^{-2}
CPN2	2.992	1.68×10^{-2}
ADH4	2.967	3.13×10^{-13}
ZPLD1	2.889	1.04×10^{-12}
C17orf99	2.805	9.45×10^{-3}

PDZK1IP1	2.801	1.58×10^{-3}
GABRG1	2.748	8.24×10^{-7}
XPNPEP2	2.740	2.25×10^{-2}
TBX4	2.724	3.11×10^{-2}
SLC14A2	2.598	4.38×10^{-23}
SUSD2	2.576	2.99×10^{-2}
SAA1	2.569	9.69×10^{-3}
SLC10A1	2.513	1.47×10^{-2}
AGXT2	2.482	2.21×10^{-2}
CPS1	2.439	1.89×10^{-2}
ODAPH	2.366	1.32×10^{-2}
SOWAHD	2.325	2.95×10^{-2}
PCP4L1	2.287	2.85×10^{-3}
KNG1	2.260	6.81×10^{-3}
SULT2A1	2.247	4.92×10^{-2}
P2RY4	2.151	1.72×10^{-2}
CSF3R	2.141	4.44×10^{-2}
CHP2	2.112	4.11×10^{-2}
SLC6A20	2.104	2.84×10^{-2}
SLC5A8	2.081	1.09×10^{-3}
C4BPA	2.072	2.56×10^{-2}
ORM1	2.069	1.99×10^{-2}
TNFRSF1B	2.055	3.36×10^{-2}
MAT1A	2.040	5.75×10^{-3}
PRSS36	2.034	1.96×10^{-3}
ABCC2	2.029	4.20×10^{-2}
ORM2	2.028	1.54×10^{-2}
KCNE3	2.024	1.12×10^{-3}
THPO	2.019	6.21×10^{-3}
SLC38A5	2.010	1.38×10^{-2}
ABI3BP	1.999	2.66×10^{-2}
APELA	1.993	3.52×10^{-2}
NPL	1.970	8.18×10^{-3}
CASP5	1.963	4.21×10^{-2}
XKRX	1.960	2.12×10^{-2}
CD244	1.956	1.90×10^{-2}
CTSV	1.928	3.94×10^{-2}
ATP6V0A4	1.914	3.28×10^{-2}

SLC46A3	1.913	2.26×10^{-2}
DKK4	1.908	2.20×10^{-2}
ERVH48-1	1.904	1.53×10^{-3}
AIRE	1.903	1.75×10^{-2}
BST2	1.901	2.70×10^{-2}
ABCA10	1.871	5.23×10^{-3}
DEFB1	1.869	2.77×10^{-2}
APOB	1.867	2.34×10^{-2}
TPH1	1.863	1.07×10^{-14}
IL22RA1	1.862	4.17×10^{-2}
LYPD2	1.850	2.09×10^{-4}
P2RX1	1.848	2.10×10^{-2}
SLPI	1.848	1.95×10^{-2}
MUC17	1.847	2.13×10^{-2}
SLC23A1	1.833	3.24×10^{-2}
APOD	1.823	1.79×10^{-2}
PAPPA2	1.822	2.35×10^{-11}
SAA2	1.818	4.11×10^{-2}
CTXND1	1.816	7.48×10^{-3}
AOC1	1.816	1.57×10^{-3}
G0S2	1.815	4.81×10^{-2}
ALX1	1.809	1.02×10^{-2}
KCNA4	1.808	7.92×10^{-5}
MRO	1.795	3.08×10^{-2}
PSAPL1	1.795	4.81×10^{-3}
CPA2	1.793	4.15×10^{-2}
KCNJ13	1.791	2.43×10^{-3}
CASP1	1.787	1.47×10^{-2}
CCR1	1.755	6.84×10^{-3}
KCNJ12	1.735	2.59×10^{-2}
LRRC19	1.697	2.95×10^{-2}
CKM	1.694	2.14×10^{-6}
WIPF3	1.685	1.93×10^{-3}
CREG2	1.652	4.71×10^{-2}
SLC15A1	1.641	3.46×10^{-2}
CSF2RA	1.639	2.30×10^{-6}
PIGR	1.627	2.11×10^{-2}
TXNIP	1.620	9.37×10^{-4}

AKR1D1	1.617	2.00×10^{-4}
ANPEP	1.611	4.13×10^{-2}
CD3G	1.610	3.91×10^{-2}
MUCL3	1.610	8.54×10^{-7}
GPA33	1.603	2.64×10^{-2}
NOX1	1.600	1.20×10^{-2}
ZG16	1.579	4.62×10^{-2}
RGPD1	1.574	7.35×10^{-3}
LY75	1.571	3.22×10^{-3}
RUBCNL	1.571	3.43×10^{-2}
OLR1	1.557	4.53×10^{-3}
NAGS	1.554	3.14×10^{-2}
C3	1.545	6.49×10^{-7}
MBNL3	1.545	1.10×10^{-2}
FAM3D	1.545	2.80×10^{-2}
SHBG	1.535	2.65×10^{-2}
PPP1R3G	1.533	1.48×10^{-2}
CLDN19	1.484	3.19×10^{-2}
P2RY6	1.470	3.80×10^{-2}
PRDM1	1.470	6.03×10^{-3}
CCRL2	1.467	4.75×10^{-3}
MUC3A	1.457	2.49×10^{-2}
PAH	1.438	2.29×10^{-5}
CEACAM7	1.428	4.98×10^{-3}
ASS1	1.412	2.42×10^{-3}
ACE2	1.406	5.43×10^{-3}
GBP2	1.405	6.12×10^{-7}
SLC51B	1.399	3.25×10^{-2}
HYAL1	1.399	2.23×10^{-2}
SLC27A2	1.384	4.44×10^{-3}
ADH1A	1.382	4.80×10^{-9}
RETREG1	1.379	4.29×10^{-3}
MYEOV	1.375	6.28×10^{-3}
CTSA	1.372	4.19×10^{-2}
BATF2	1.371	3.46×10^{-2}
TNFSF10	1.370	1.80×10^{-2}
MFSD2A	1.368	2.69×10^{-2}
APOA2	1.363	1.17×10^{-2}

KCNJ5	1.356	1.44 x 10^{-3}
MUC13	1.351	4.74 x 10^{-2}
ROS1	1.347	7.40 x 10^{-4}
PBLD	1.345	1.14 x 10^{-2}
C1orf115	1.337	5.54 x 10^{-3}
GPAT3	1.337	3.93 x 10^{-3}
HSD3B1	1.327	7.69 x 10^{-3}
SLC16A10	1.326	6.62 x 10^{-3}
MRGPRF	1.325	2.95 x 10^{-2}
ACTN3	1.325	1.87 x 10^{-2}
KYNU	1.322	4.09 x 10^{-4}
SLC23A3	1.307	2.43 x 10^{-3}
DUSP9	1.305	1.49 x 10^{-2}
CHST13	1.302	4.63 x 10^{-2}
SLC2A2	1.292	8.84 x 10^{-3}
ABCG2	1.287	7.98 x 10^{-3}
SLC18A2	1.286	2.28 x 10^{-5}
RAB42	1.285	5.59 x 10^{-3}
CEL	1.283	3.28 x 10^{-2}
CD302	1.273	4.45 x 10^{-3}
NCR3LG1	1.271	1.14 x 10^{-2}
SLC7A8	1.270	1.21 x 10^{-2}
STRIP2	1.267	3.40 x 10^{-2}
COMP	1.263	5.95 x 10^{-6}
KBTBD12	1.262	2.95 x 10^{-2}
FAM20A	1.261	1.14 x 10^{-2}
TIFA	1.259	8.10 x 10^{-3}
C10orf95	1.256	3.78 x 10^{-2}
TRPA1	1.255	3.48 x 10^{-3}
SULT1B1	1.254	1.44 x 10^{-2}
CADM2	1.247	1.53 x 10^{-8}
SLCO4C1	1.242	4.99 x 10^{-3}
SLC35G1	1.240	1.10 x 10^{-2}
CYP8B1	1.237	2.55 x 10^{-3}
MYO1A	1.230	2.19 x 10^{-2}
GGT5	1.228	2.34 x 10^{-2}
SULT1C2	1.224	4.09 x 10^{-3}
MOCOS	1.221	3.72 x 10^{-2}

CYP2W1	1.221	1.19 x 10^{-2}
ADAMTSL4	1.215	1.36 x 10^{-2}
FUT2	1.213	3.11 x 10^{-3}
ITPKA	1.213	3.96 x 10^{-2}
GIPC2	1.211	1.43 x 10^{-2}
CYP51A1	1.208	1.01 x 10^{-2}
TMEFF2	1.204	4.88 x 10^{-11}
PKD1L2	1.202	8.26 x 10^{-3}
CA2	1.198	2.30 x 10^{-3}
TPP1	1.194	8.40 x 10^{-3}
TMEM220	1.194	4.68 x 10^{-2}
LTF	1.192	3.26 x 10^{-4}
SLC31A2	1.192	6.21 x 10^{-3}
FABP1	1.186	1.56 x 10^{-2}
MUC12	1.186	2.87 x 10^{-3}
SPX	1.173	2.19 x 10^{-2}
XYLB	1.171	4.13 x 10^{-2}
ADGRF1	1.170	2.93 x 10^{-4}
ADGRG7	1.165	3.65 x 10^{-2}
KCNS1	1.159	6.04 x 10^{-3}
EPHX4	1.152	3.13 x 10^{-3}
EBI3	1.152	5.34 x 10^{-3}
CD8B	1.149	1.36 x 10^{-2}
MSMO1	1.146	1.02 x 10^{-2}
CDH22	1.144	1.26 x 10^{-2}
TRPV3	1.141	5.91 x 10^{-4}
CYP27A1	1.141	2.92 x 10^{-2}
ALPK1	1.140	1.13 x 10^{-2}
GUCY2C	1.136	1.98 x 10^{-2}
CREB3L3	1.130	2.34 x 10^{-2}
CEBPA	1.126	2.21 x 10^{-2}
IHH	1.126	1.45 x 10^{-2}
PIP5K1B	1.125	8.48 x 10^{-3}
BNIP5	1.125	2.48 x 10^{-2}
IRAG2	1.123	4.37 x 10^{-2}
GK	1.114	2.85 x 10^{-2}
LGMN	1.111	2.08 x 10^{-2}
IL1R2	1.111	2.82 x 10^{-2}

MST1R	1.110	8.71×10^{-3}
CYP4V2	1.109	2.38×10^{-3}
F2	1.108	3.54×10^{-2}
LPGAT1	1.104	6.76×10^{-3}
KLHL13	1.104	4.52×10^{-2}
MAB21L3	1.103	4.39×10^{-2}
DHRS11	1.097	2.86×10^{-2}
STEAP2	1.090	6.70×10^{-6}
CRYBG1	1.088	3.67×10^{-3}
GATM	1.087	4.56×10^{-2}
FLVCR2	1.085	3.07×10^{-2}
JPH1	1.083	2.34×10^{-2}
TTC22	1.083	2.02×10^{-2}
MGST1	1.077	1.43×10^{-2}
CDC42EP2	1.076	3.71×10^{-4}
CDX1	1.073	3.98×10^{-2}
GPLD1	1.070	3.92×10^{-3}
PRSS1	1.069	3.80×10^{-2}
NR1H4	1.068	4.33×10^{-2}
GABRB1	1.064	1.92×10^{-2}
VDR	1.064	1.17×10^{-2}
DLX3	1.062	3.47×10^{-2}
IFIT3	1.061	2.98×10^{-2}
ACE	1.060	3.67×10^{-2}
SMIM31	1.054	2.98×10^{-2}
FGA	1.053	4.10×10^{-2}
PCDHA10	1.049	1.70×10^{-2}
CASP10	1.047	6.43×10^{-4}
CLDN15	1.044	8.48×10^{-3}
TMEM92	1.043	1.23×10^{-2}
TMEM38B	1.041	3.19×10^{-2}
CARD14	1.041	3.70×10^{-2}
ALDH3B1	1.039	4.27×10^{-2}
TRIM14	1.039	2.18×10^{-2}
TMEM144	1.036	8.38×10^{-3}
B3GALT5	1.034	6.77×10^{-3}
CA4	1.033	1.10×10^{-4}
ALDH5A1	1.032	6.38×10^{-3}

VIPR1	1.032	3.42×10^{-3}
TFPI	1.027	1.47×10^{-7}
STOM	1.026	5.08×10^{-3}
MAF	1.026	2.30×10^{-2}
S100P	1.025	3.42×10^{-3}
BMP2K	1.025	3.19×10^{-4}
ERAP2	1.024	2.03×10^{-3}
PRR15	1.021	7.14×10^{-3}
GALNT5	1.020	2.28×10^{-3}
COBLL1	1.014	6.26×10^{-3}
FGF10	1.013	2.20×10^{-2}
PCSK5	1.009	2.42×10^{-2}
EDA	1.005	1.93×10^{-3}
IL2RG	1.001	4.31×10^{-2}
SGK3	1.001	2.53×10^{-2}

ESM Figures

ESM Figure 1

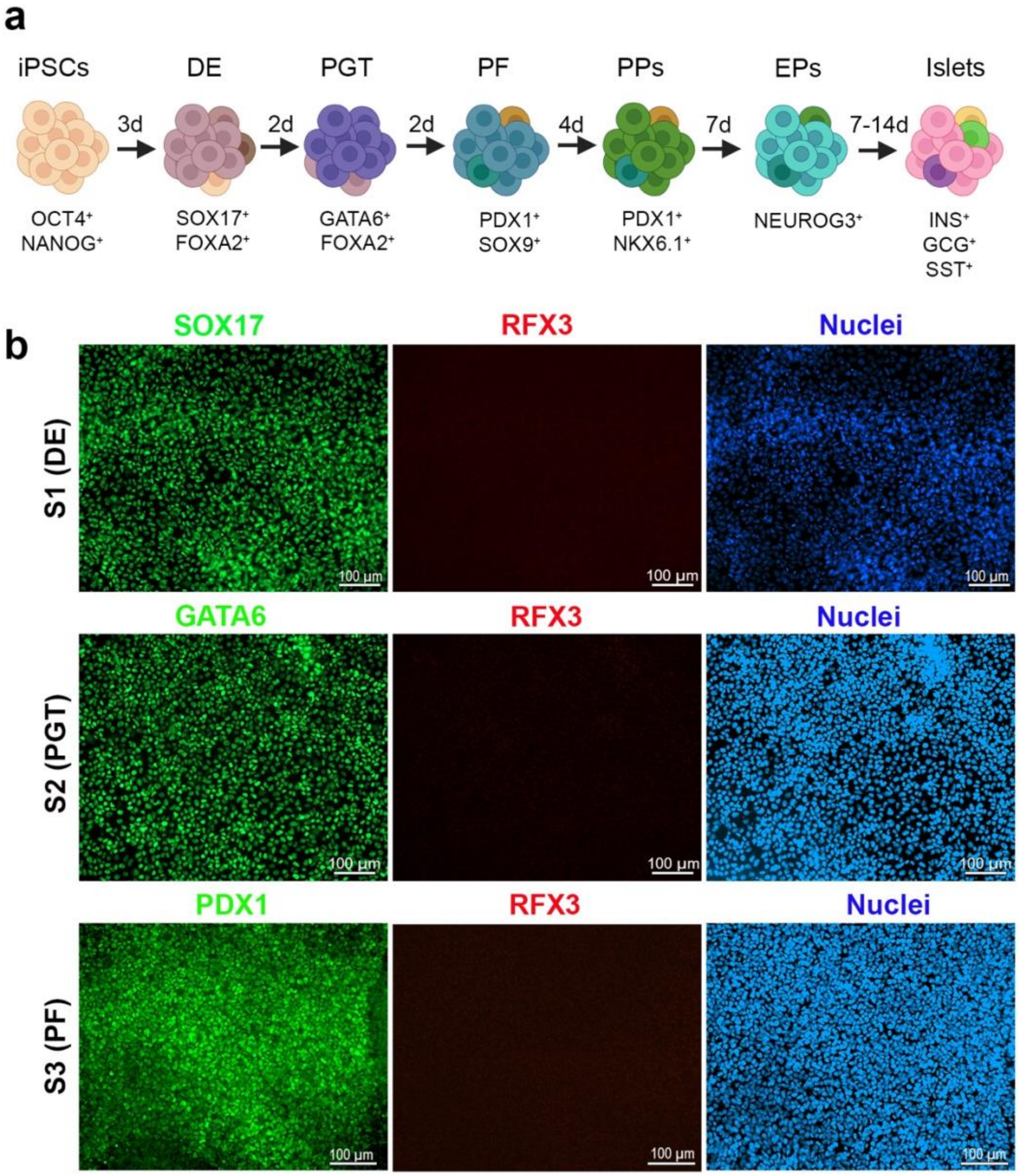

ESM Figure 1: RFX3 expression at different stages during iPSC differentiation into pancreatic islets. (**a**) Schematic representation of the *in vitro* pancreatic islet differentiation protocol (created with BioRender). (**b**) Immunofluorescence images for co-expression of RFX3 with definitive endoderm (SOX17), primitive gut tube (GATA6) and posterior foregut (PDX1) markers during early stages of iPSC differentiation to islets. Note the absence of RFX3 (red) expression in early stages of islet differentiation. Scale bar = 100 μm.

ESM Figure 2

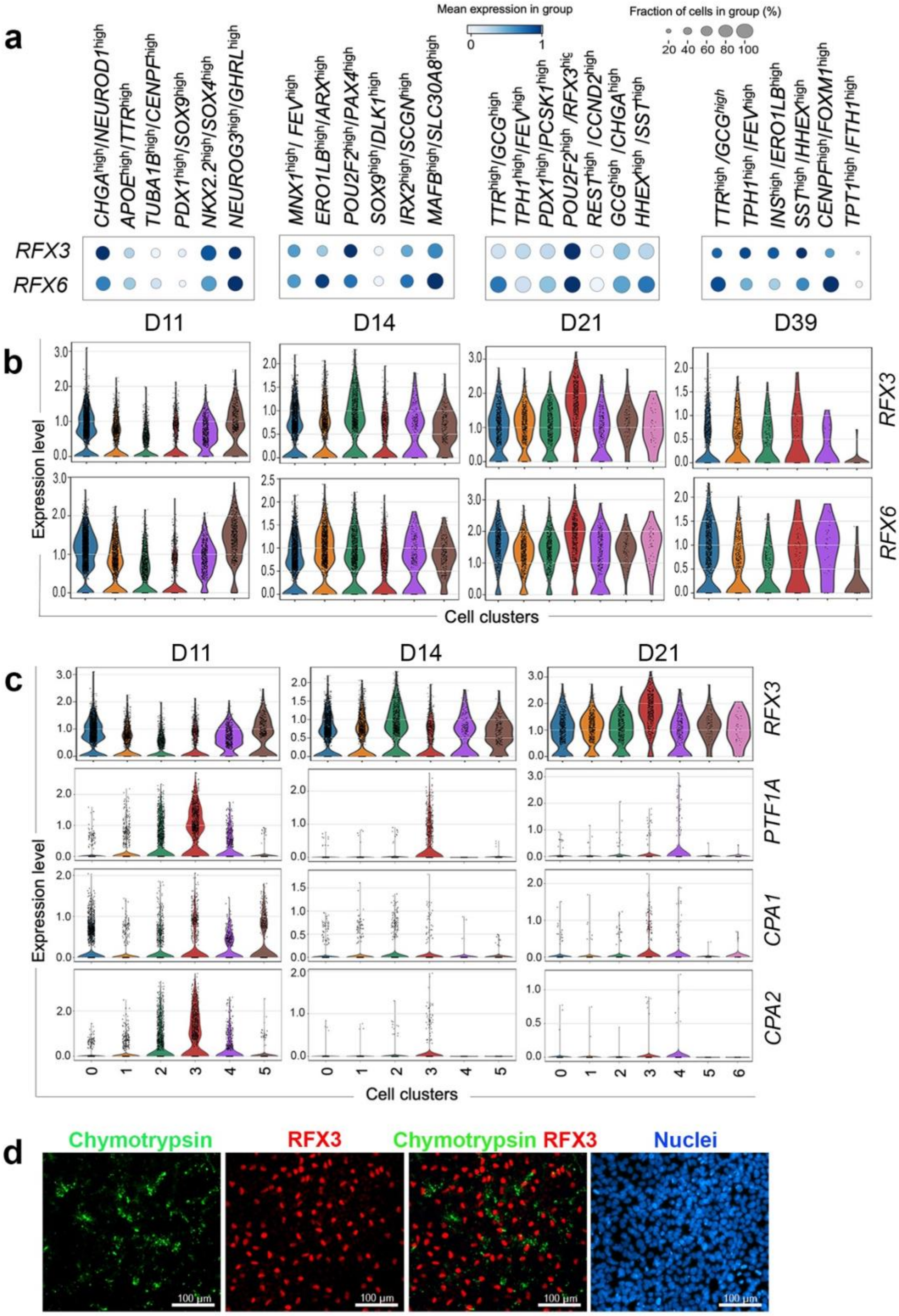

ESM Figure 2: (**a**) Dot plots demonstrating *RFX3* and *RFX6* expression in various cell clusters determined by single-cell RNA-sequencing. Expression level in each cluster is scaled based on percentages of cells expressing *RFX3* (dot size) and mean expression (colour intensity) of the gene. Dot plots are presented for day 11 (D11), day 14 (D14), day 21 (D21), and day 39 (D39) of hESC differentiation. (**b**) Violin plots depicting expression pattern of *RFX3* and *RFX6* across various cell clusters at D11, D14, D21, and D39. (**c**) Violin plots depicting expression pattern of *RFX3* and exocrine markers, *PTF1A*, *CPA1*, and *CPA2* across cell clusters at D11, D14, and D21. (**d**) immunostaining images showing the expression of exocrine marker, chymotrypsin, and RFX3 in iPSC-derived pancreatic progenitors.

ESM Figure 3

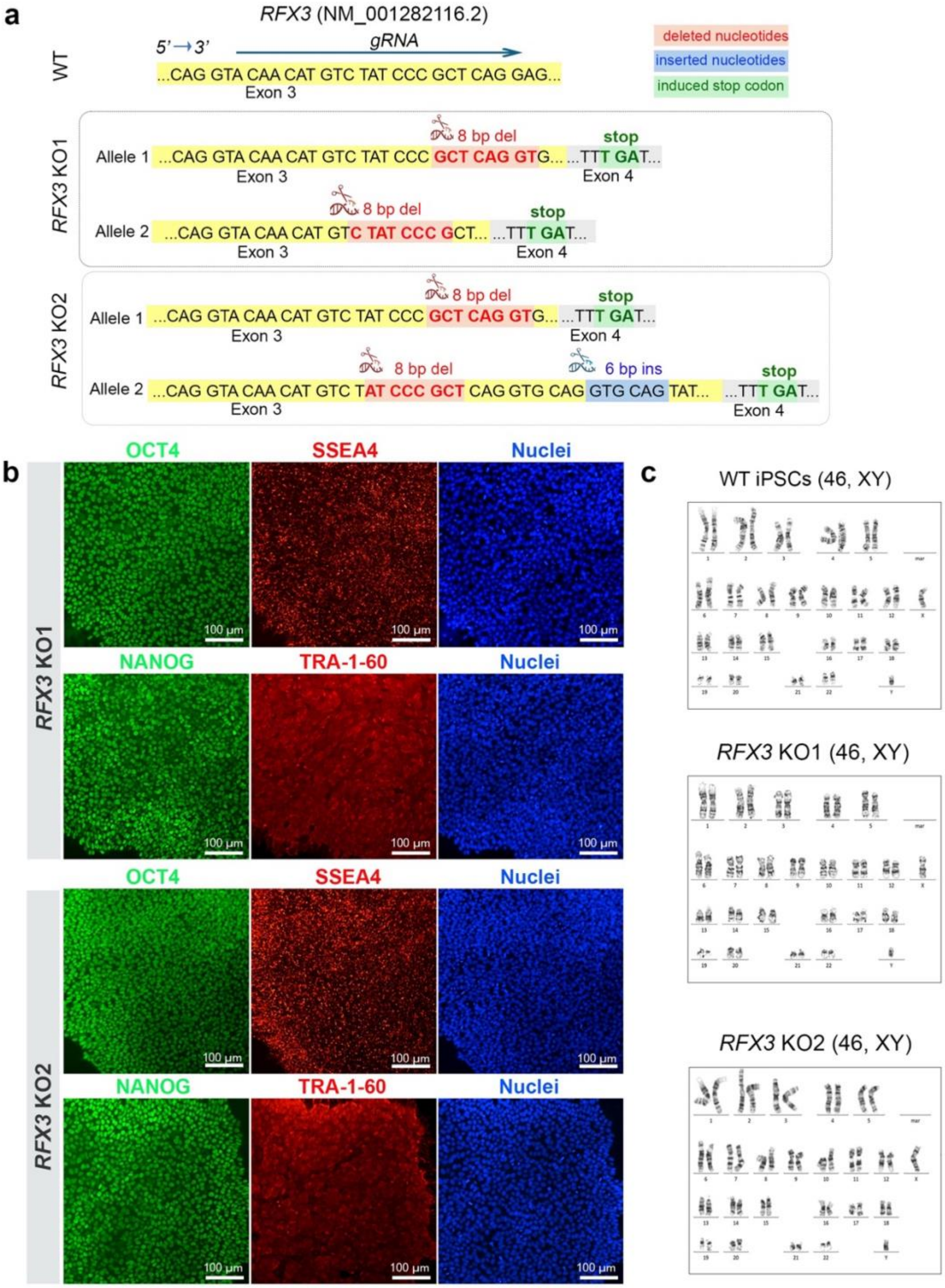

ESM Figure 3: Validation and characterization of *RFX3* KO iPSC lines. (**a**) Sanger sequencing results demonstrating the edits and deletions in the *RFX3* gene, which resulted in the formation of stop codons. (**b**) Immunofluorescence images for expression of pluripotency markers for *RFX3* KO iPSCs showing their high levels in undifferentiated cells, and (**c**) karyotype analysis of *RFX3* KO clones showing normal number of chromosomes, similar to WT. Scale bar = 100 µm.

ESM Figure 4

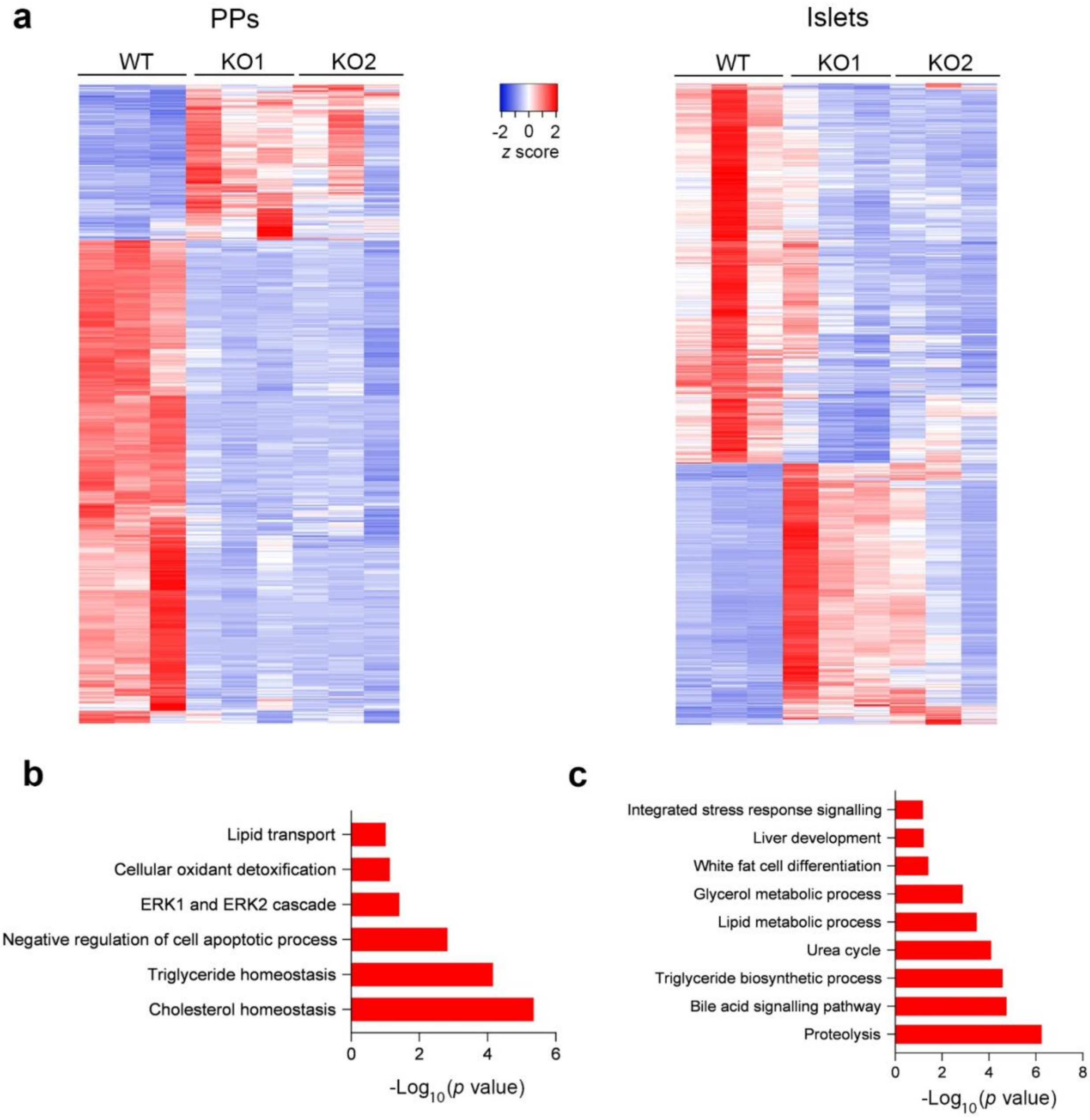

ESM Figure 4: Transcriptome profiling alterations associated with *RFX3* loss in pancreatic progenitors (PPs) and islets. (**a**) A general clustering heatmap of differentially expressed genes (DEGs) in PPs and islets derived from WT and *RFX3* KO iPSCs. Selected gene ontology pathways associated with upregulated DEGs in PPs (**b**) and islets (**c**).

ESM Figure 5

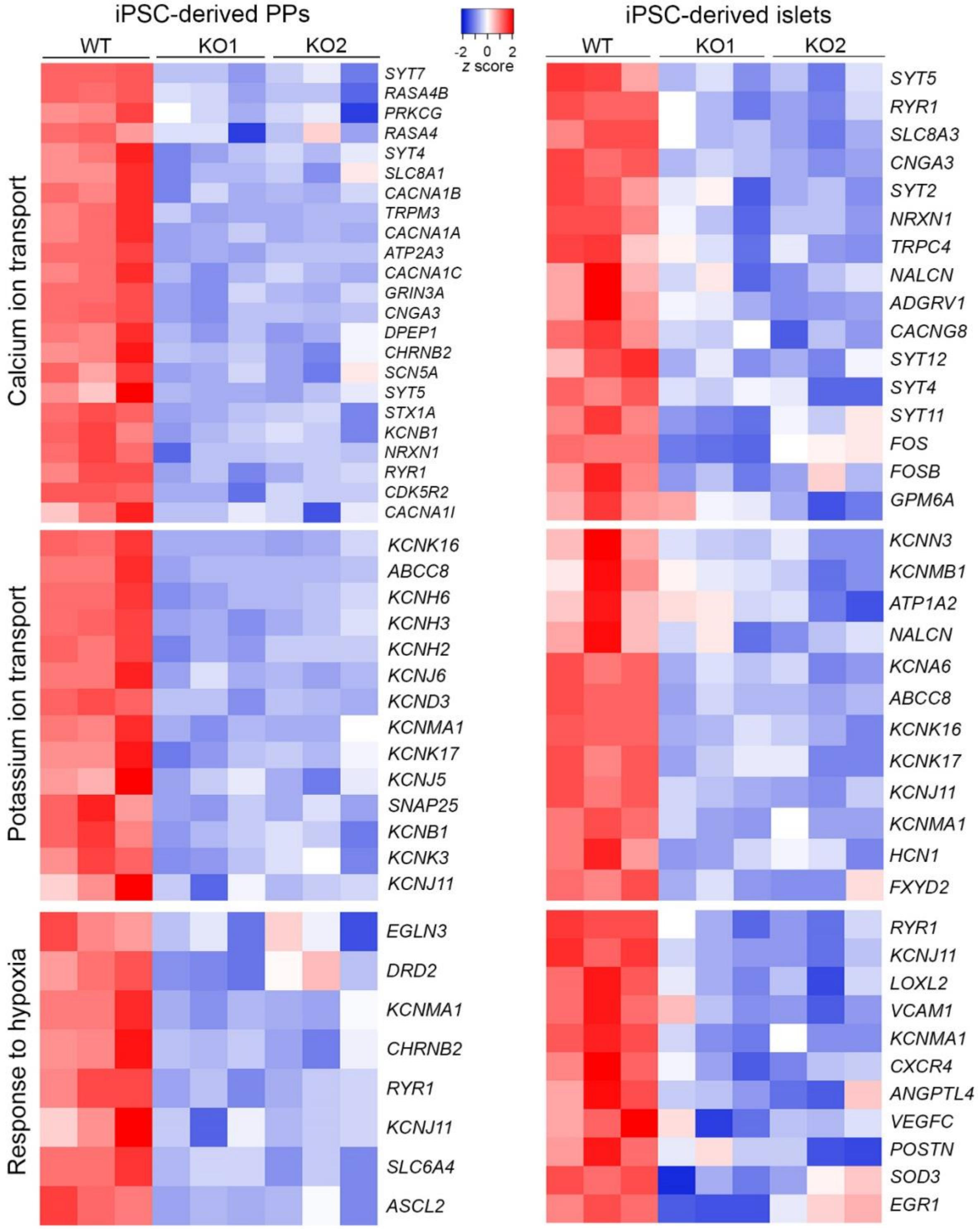

ESM Figure 5: Heatmaps of downregulated DEGs highlighted enrichment in pathways related to calcium and potassium ion transport, as well as response to hypoxia, in *RFX3* KO PPs and islets, comparted to WT controls.

ESM Figure 6

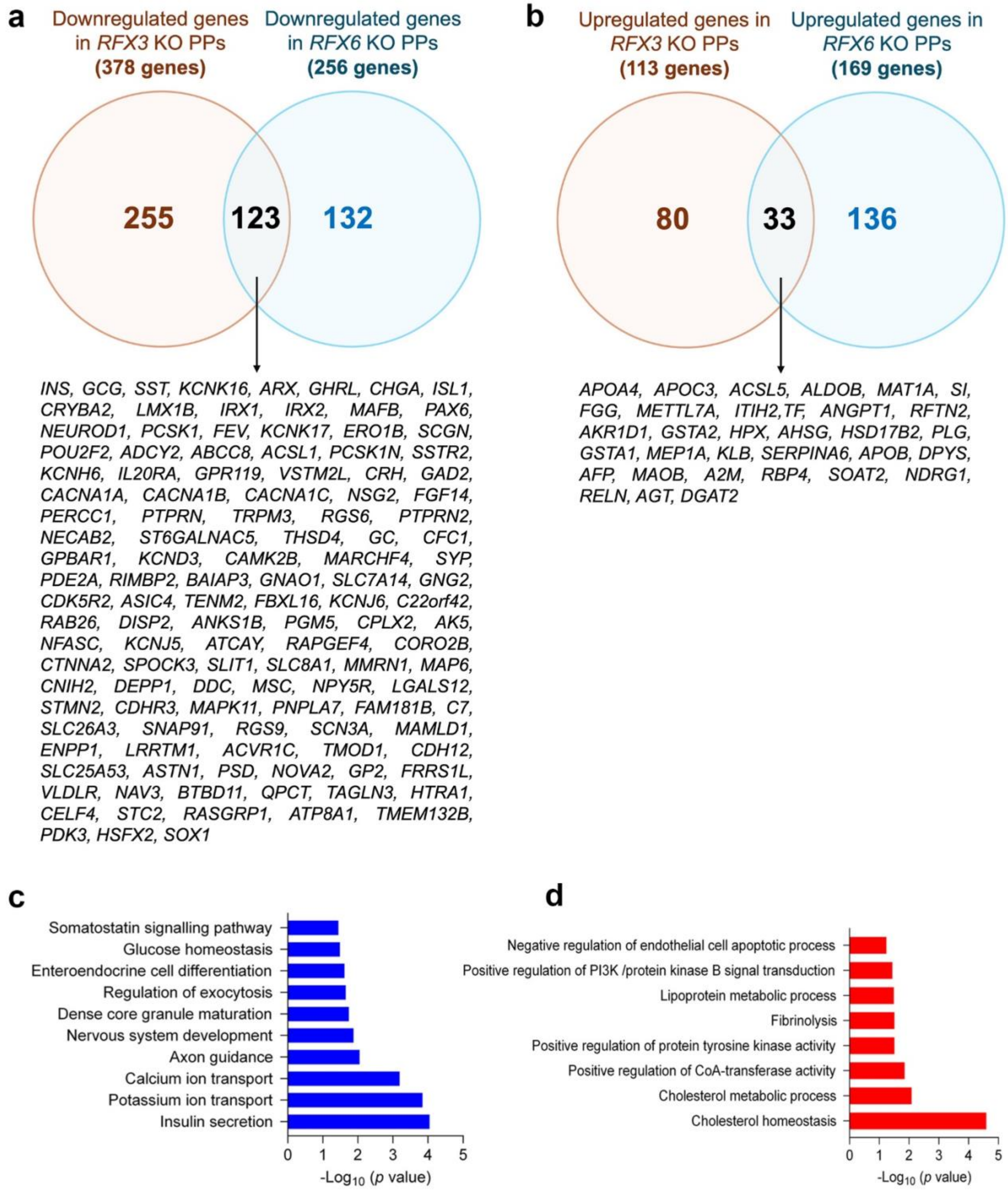

ESM Figure 6: Common differentially expressed genes (DEGs) in pancreatic progenitors lacking *RFX3* and *RFX6*. (**a**) 123 DEGs were commonly downregulated in both *RFX3* KO and *RFX6* KO

pancreatic progenitors (PPs) compared to WT PPs, representing 32.5% of the downregulated genes in *RFX3* KO PPs, while 255 genes (67.46%) were downregulated in *RFX3* KO PPs, but not in *RFX6* KO PPs. (**b**) A total of 33 DEGs were commonly upregulated in both *RFX3* KO and *RFX6* KO PPs compared to WT PPs, representing 29.2% of the upregulated DEGs in *RFX3* KO PPs. 80 out of 113 genes (70.79%) were specially upregulated in *RFX3* KO PPs, but not in *RFX6* KO PPs compared to WT PPs. Gene ontology of significantly enriched biological processes in commonly downregulated (**c**) and commonly upregulated (**d**) DEGs.

ESM Figure 7

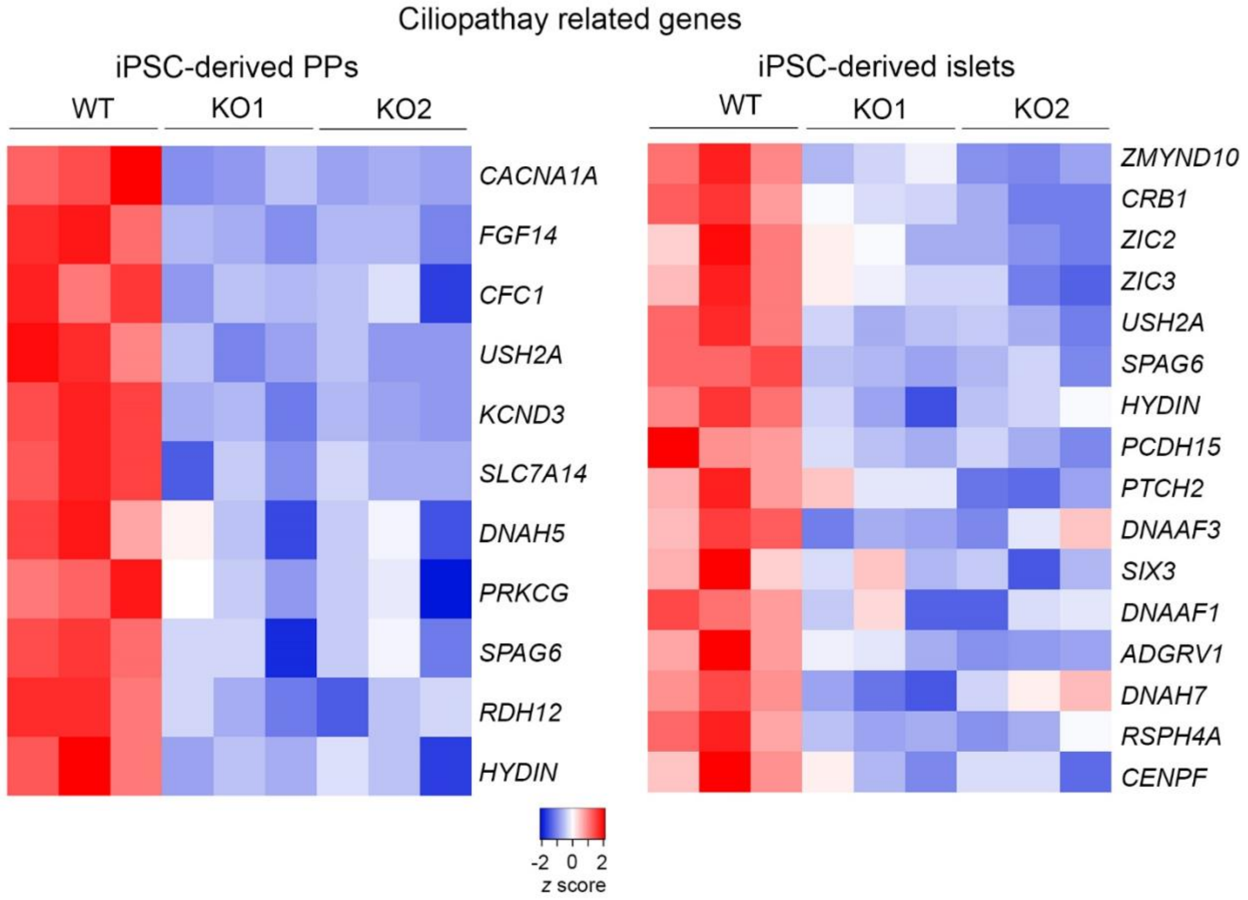

ESM Figure 7: Alterations in the expression of ciliopathy-related genes obtained from CiliaMiner database in pancreatic progenitors and islets lacking *RFX3*.

ESM Fig. 8

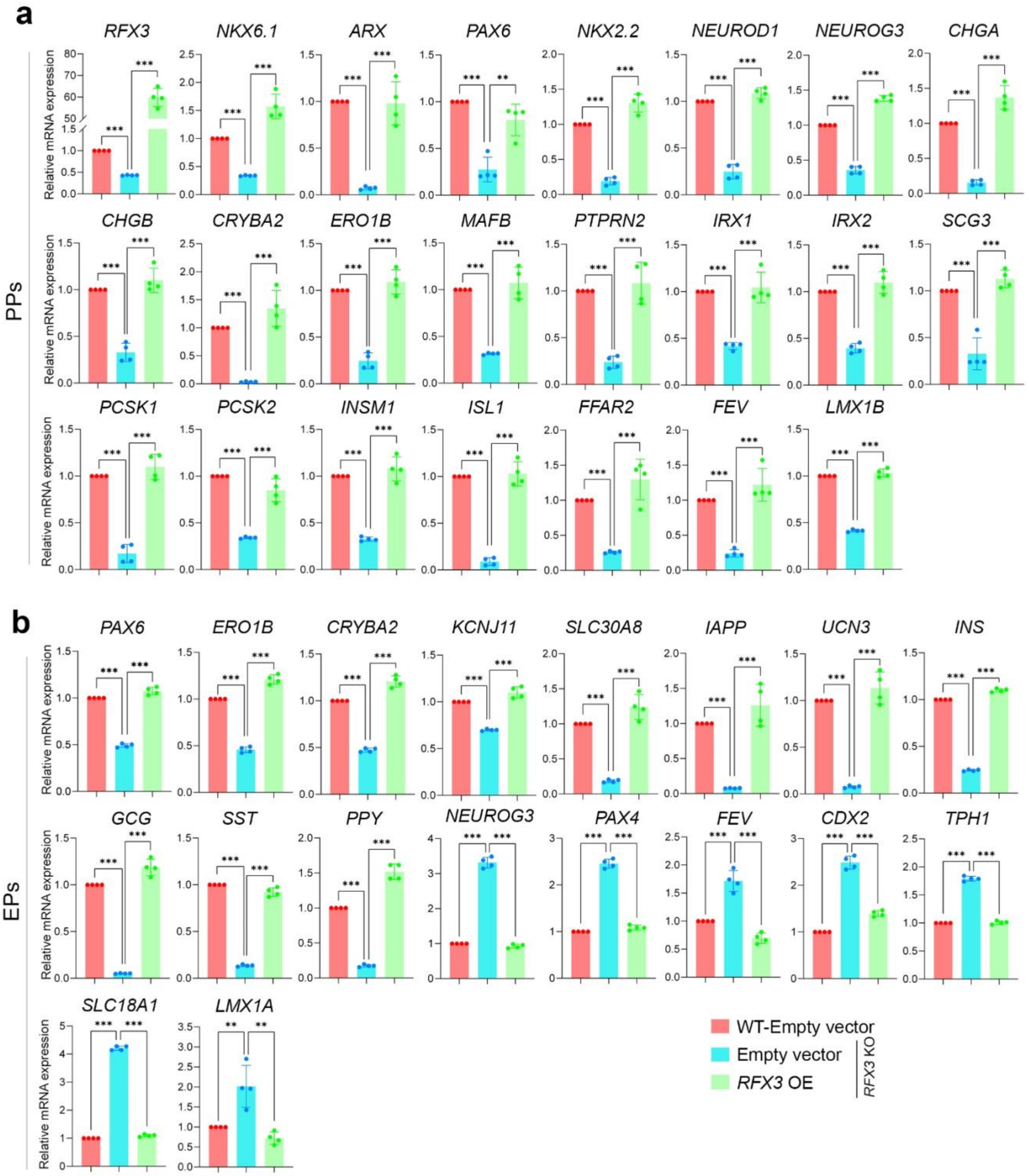

ESM Figure 8: RFX3 overexpression rescues the expression of dysregulated genes in pancreatic cell lacking *RFX3*. (**a**) RT-qPCR analysis for the expression of pancreatic endocrine genes

including, *RFX3, NKX6.1, ARX, PAX6, NKX2.2, NEUROD1, NEUROG3, CHGA, CHGB, CRYBA2, ERO1B, MAFB, PTPRN2, IRX1, IRX2, SCG3, PCSK1, PCSK2, INSM1, ISL1, FFAR2, FEV* and *LMX1B* in PPs derived from RFX3 KO iPSCs and WT-iPSCs, 48 h following ectopic expression of RFX3 ($n=4$). (**b**) RT-qPCR analysis for the expression of pancreatic endocrine and enterochromaffin genes including, *NEUROG3, PAX6, ERO1B, CRYBA2, KCNJ11, SLC30A8, IAPP, UCN3, INS, GCG, SST, PPY, PAX4, FEV, CDX2, TPH1, SLC18A1,* and *LMX1A* in EPs derived from RFX6 KO iPSCs and WT-iPSCs, 120 h following ectopic expression of RFX3 ($n=4$). Data are represented as mean ± SD; $^{**}p < 0.01$, $^{***}p < 0.001$.
